# Supplementary material for: A photocatalytic redox cycle over a polyimide catalyst drives efficient solar-to-H2O2 conversion
Source: Nat Commun. 2024 Jun 22;15:5316. doi: 10.1038/s41467-024-49663-6 (PMC11535368; doi:10.1038/s41467-024-49663-6)
Supplement: Supplementary file 1 — Supplementary Information [file 41467_2024_49663_MOESM1_ESM.pdf]

Supplementary Information for

A photocatalytic redox cycle over a polyimide catalyst drives efficient solar-to-H_2O_2 conversion

Wenwen Chi[1,4], Yuming Dong[1,4✉], Bing Liu[1], Chengsi Pan[1], Jiawei Zhang[1], Hui Zhao[1], Yongfa Zhu[2,✉], Zeyu Liu[3]

Affiliations

[1]*International Joint Research Center for Photoresponsive Molecules and Materials, Key Laboratory of Synthetic and Biological Colloids, School of Chemical and Material Engineering, Jiangnan University, Wuxi 214122, China.*

[2]*Department of Chemistry, Tsinghua University, Beijing 100084, China*.

[3]*School of Environmental and Chemical Engineering, Jiangsu University of Science and Technology, Zhenjiang 212100, China.*

[4]*These authors contributed equally: Wenwen Chi, Yuming Dong.*

[✉]e-mail: *zhuyf@tsinghua.edu.cn; dongym@jiangnan.edu.cn*

Table of Contents

Section 1. Methods **4**

1.1 Characterization 4

1.2 General methods 5

Photocatalytic H_2O_2 production 5

In situ DRIFTS Characterization 5

In situ XPS Characterization 6

In situ Raman Characterization 6

Section 2. Photocatalytic performance for BD-TPB **7**

2.1 Synthesis of PI-BD-TPB aerogel 7

2.2 FTIR spectra of sol-gel-thermal imidization process 8

2.3 Photocatalytic activity experiment 9

2.4 The stability of BD-TPB photocatalyst 11

2.5 The SCC of BD-TPB photocatalyst 14

2.6 Photocatalytic decomposition of hydrogen peroxide 15

2.7 Practical performance tests of BD-TPB photocatalyst 16

2.8 Comparison of photocatalytic H_2O_2 activity 19

Section 3. Characterizations of PI-BD-TPB photocatalyst **20**

3.1 XPS spectra of BD-TPB 20

3.2 GPC spectra of PAA 21

3.3 SEM analysis of BD-TPB 22

3.4 TEM analysis of BD-TPB 23

3.5 XRD analysis of BD-TPB 24

3.6 Nitrogen isotherm and pore size distribution of BD-TPB 24

3.7 Mercury intrusion measurement of BD-TPB................................. 25

3.8 TGA analysis of BD-TPB... 25

3.9 Chemical stability... 26

3.10 Zeta potential analysis 27

3.11 Contact angle measurement of BD-TPB 27

3.12 Additional Characterizations of BD-TPB 28

Section 4. PI-BD-TPB aerogel with reductive carbonyl groups.......... 32

4.1 Theoretical simulations 32

4.2 Photoelectrochemical measurements... 33

4.3 EPR measurement for superoxide radical...................................... 38

4.4 Fluorescence measurement.. 39

Section 5. The mechanism of water oxidation 40

Section 6. The mechanism of photocatalytic redox cycle..................... 45

6.1 Isotopic labeling experiment 45

6.2 *In situ* FTIR analysis 47

6.3 Elemental analysis.. 47

6.5 H_2O_2 production on various PI photocatalysts with carbonyl group48

Section 7. Theoretical simulations and computation 55

Section 8. Supplementary References 61

Section 1. Methods

1.1 Characterization

The crystal structure was characterized via powder X-ray diffraction (XRD, D8 Bruker, German) with a Cu Ka (1.5418 Å) monochromator at 40 kV and 40 mA. The morphology and structure of the materials were characterized via field-emission scanning electron microscopy (SEM, S-4800, Hitachi, Japan) and transmission electron microscopy (TEM, JEOL 2100plus, Japan). X-ray photoelectron spectroscopy (XPS) is carried out to determinate the surface element (XPS, Axis Supra instrument, Kratos, UK). The XPS spectrum was calibrated using the C 1s peak (284.8 eV) to eliminate the charge effect. The UV-vis DRS adsorption was recorded by ultraviolet-visible (UV-vis) spectroscopy using a UV-3600 plus ultraviolet-visible spectrophotometer (Shimadzu, Japan). The contact angles of all the materials were obtained using the OCA 40 instrument (Dataphysics, German). The Brunauer-Emmett-Teller (BET) specific surface area of the sample was measured by N_2 adsorption at 77 K with ASAP2020 (Micromeritics, USA). Electron paramagnetic resonance (EPR) spectroscopy was used to detect the hydroxyl radical and superoxide radical on EMXPLUS (Bruker, German). The liquid product was analyzed by ^1H nuclear magnetic resonance (NMR) experiments using an AVANCE III HD 400 MHz spectrometer (Bruker, Switzerland) at frequencies of 400 M Hz. Fourier transform infrared (FTIR) spectra were recorded on a Nicolet iS5 spectrometer (Thermo, USA) using the KBr pellet technique. Thermogravimetric analysis was performed using a Q600 simultaneous thermal analyzer (TA, USA) under N_2 in the range of 40–800 °C at a heating rate of 10 °C/min^{-1}. The C, H and N contents were measured with an elemental analyzer (Vario EL cube, Elementar, German). Gel permeation chromatography (GPC) measurement was performed on Agilent PLGPC 220 instrument with 1-methayl-2-pyrrolidinone (NMP) solution at 25 °C. Photoluminescence decay spectra were recorded on a LifeSpec II fluorescence lifetime spectrometer (Edinburgh). ^{18}O isotopic tracing in H_2O_2 was measured by Liquid Water Isotope Analyser (Los Gatos Research).

1.2 General methods

Photocatalytic H_2O_2 production

The H_2O_2 concentration was determined by a potassium titanium oxalate method [1]. 1.5 mL solution was taken from the reactor with an injector topped with a 0.22 μm filter at given time intervals, and then, mixed with 1 mL of 0.005 M potassium titanium (IV) oxalate solution. The solution then changed from transparent to yellow due to the formation of Ti peroxides. The absorbance at 400 nm was used to determine the concentration of H_2O_2 and monitored by a UV-vis spectrometer.

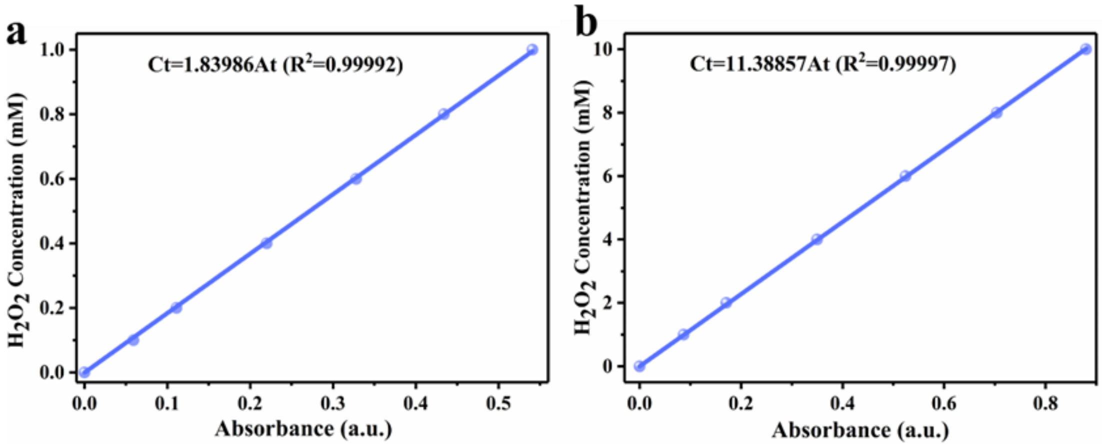

Figure S1 (a) The curve of the low concentration of H_2O_2; (b) The curve of the high concentration of H_2O_2.

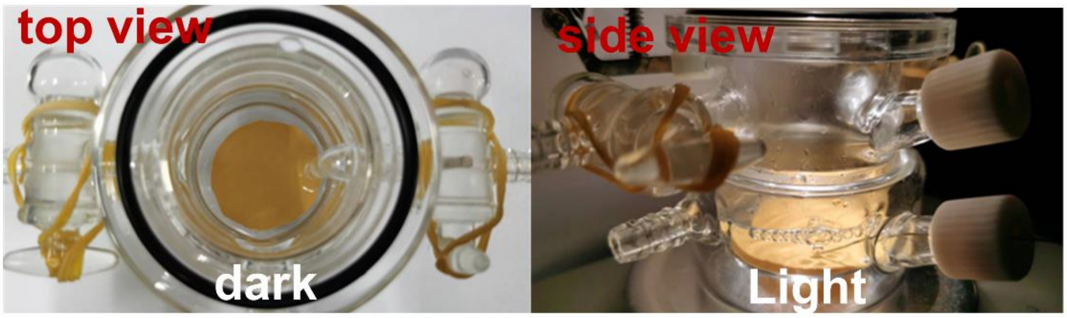

Figure S2 The photograph of photosynthesis of H_2O_2 device.

In situ DRIFTS Characterization

In situ Fourier transform infrared spectroscopy (ATR-FTIR) of HMF adsorption was performed using a Bruker V70 FTIR spectrometer with an MCT detector. The sample was loaded into a cell from HARRIC. Each spectrum was recorded with 32 scans at a resolution of 4 cm^{-1} under light irradiation. The chamber was sealed with two ZnSe windows. The sample was prepared as follows: 10 mg of power photocatalyst was dispersed in 10 mL of ethanol containing 50 μL

of Nafion by ultrasonication. 100 μL of the above slurry was put onto the disk and dried.

In situ XPS Characterization

The electronic structure changes were determined by X-ray photoelectron spectroscopy (XPS, ESCALAB 250 Xi, ThermoFisher Scientific) with 300 W Xe lamp (26.5 mW cm^{-2}).

In situ Raman Characterization

The Raman spectra was conducted on a Renishaw inVia confocal microscope, using an excitation wavelength of 785 nm, combined with 300 W Xe lamp. The O_2-saturated in 10% (v / v) 2-propanol aqueous solution. *In situ* Raman measurements were conducted at a range of 1200 to 700 cm^{-1}. Each Raman spectrum was collected during the photoreaction by every 15 min. The sample was prepared as follows: 10 mg of power photocatalyst was dispersed in 1mL of ethanol containing 50 μL of Nafion by ultrasonication.

Section 2. Photocatalytic performance for BD-TPB

2.1 Synthesis of PI-BD-TPB aerogel

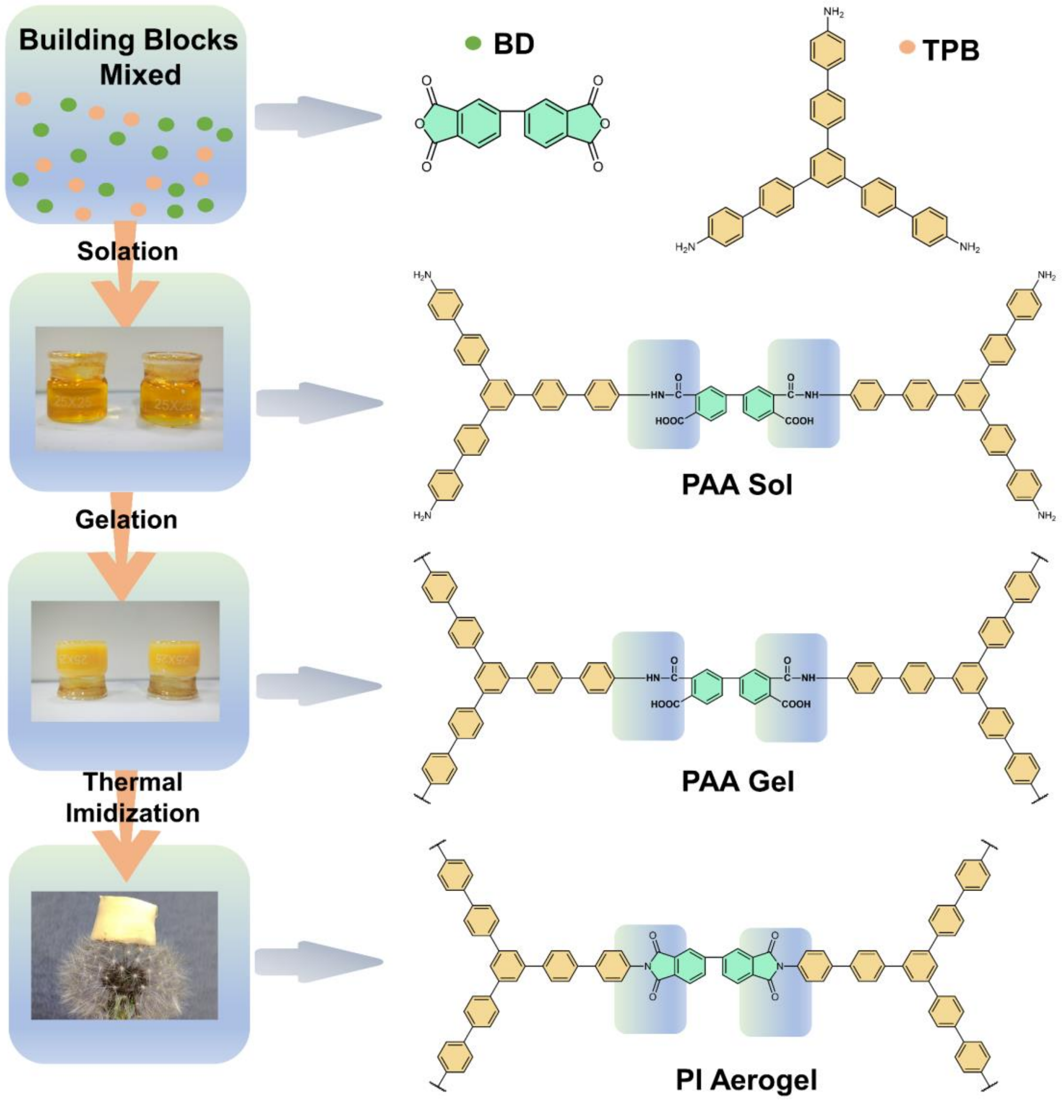

Figure S3 Schematic illustration of polyimide aerogel via sol-gel-thermal imidization route.

Polyimide aerogel was prepared via the condensation reaction of aromatic anhydride and triamines. The triangular triamines 1,3,5-tris[4-amino(1,1-biphenyl-4-yl)]-benzene (TPB) and the linear 3,3′,4,4′-biphenyltetracarboxylic dianhydride (BD) were dissolved in the solvent containing N-methylpyrrolidone (NMP) and Mesitylene. This mixture solution was then sonicated and formed to a uniform solution named as polyamic acid solution (PAA Sol). Subsequently, the highly crosslinked polymer separated from the solvents and formed polyamic acid gelatum (PAA Gel). Finally, the PAA Gel was thermally imidized to convert the residual amic acid group into the five imide rings, resulting in covalently crosslinked polyimide aerogel (PI-BD-TPB).

2.2 FTIR spectra of sol-gel-thermal imidization process

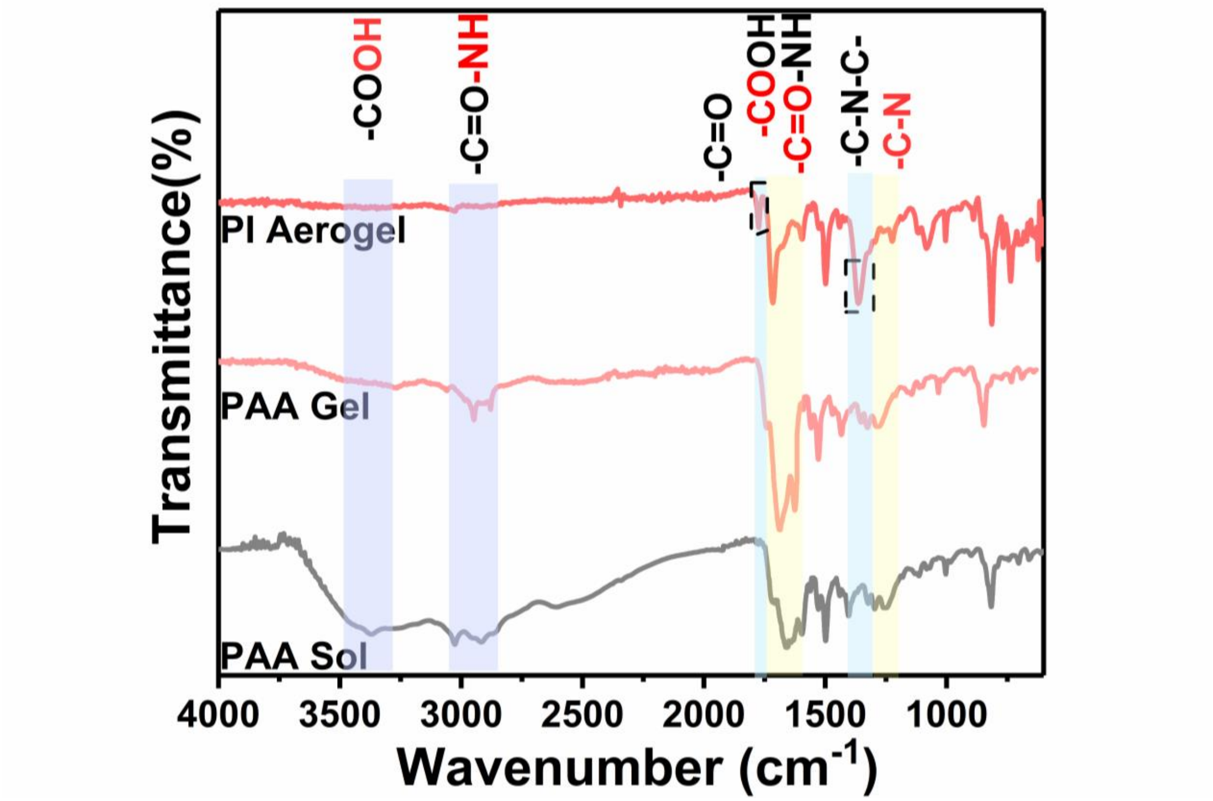

Figure S4 FTIR spectra of PAA sol, PAA gel and PI aerogel of BD-TPB.

Fourier transform infrared (FTIR) spectroscopy was employed to monitor the chemical structure change in sol-gel-thermal imidization process (**Figure S4**). One BD molecule dissolved in NMP and Mesitylene was first hydrolyzed into four -COOH groups, then -NH_3 group of TPB reacted with -COOH group forming -CONH. Characteristic peaks arisen at 1716 cm^{-1} (C=O vibrating in -COOH), 3373 cm^{-1} (O−H vibrating in -COOH), 1657 cm^{-1} (C=O vibrating in -CONH), 3020 cm^{-1} (N−H vibrating in -CONH) and 1250 cm^{-1} (C−N vibrating in -CONH) can be obviously observed in PAA Sol. After forming PAA Gel, the decrease of -COOH group was accompanied by the increase of the -CONH, indicating produced cross-linked network structure. It is clearly observed the bands at 1777.5, 1708.9 and 738.6 cm^{-1} in the curve of PI aerogel, which represented the asymmetric stretching, symmetric stretching and bending vibration of carbonyl groups, respectively. And the band at 1358.1 cm^{-1} was assigned to the stretching vibration of -C−N, evidencing the successful transformation of the PAA into PI after thermal treatment process.

2.3 Photocatalytic activity experiment

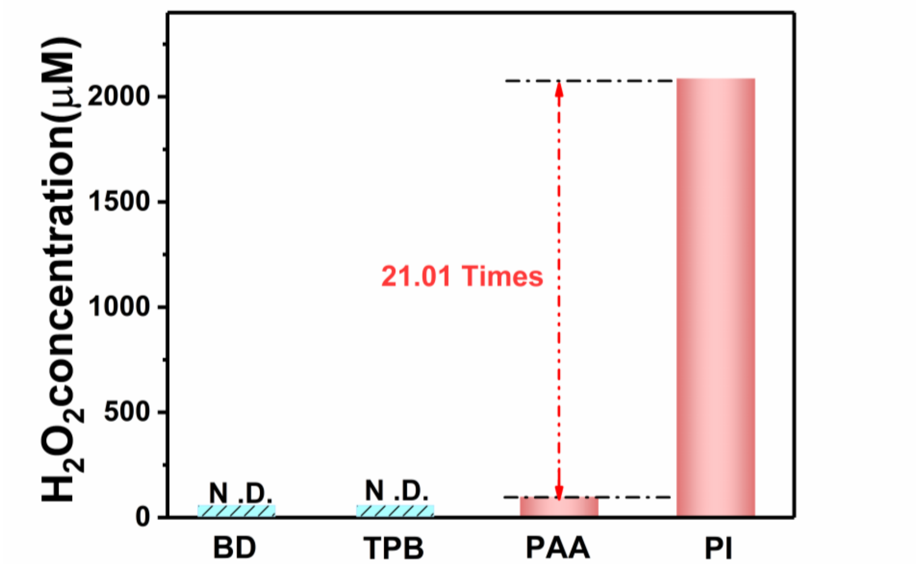

Figure S5 Comparison of photocatalytic H_2O_2 yield of PI-BD-TPB with corresponding single linker BD, TPB, and the PAA in 60 min under simulated sunlight.

The single linker BD or TPB was almost no activity in H_2O_2 photosynthesis. In the presence of final product PI-BD-TPB aerogel, the H_2O_2 concentration was almost 21 times higher that of the intermediate of PI-BD-TPB, demonstrating that five imide rings formation via sol-gel-thermal imidization route played a crucial part in photocatalytic H_2O_2 production**.**

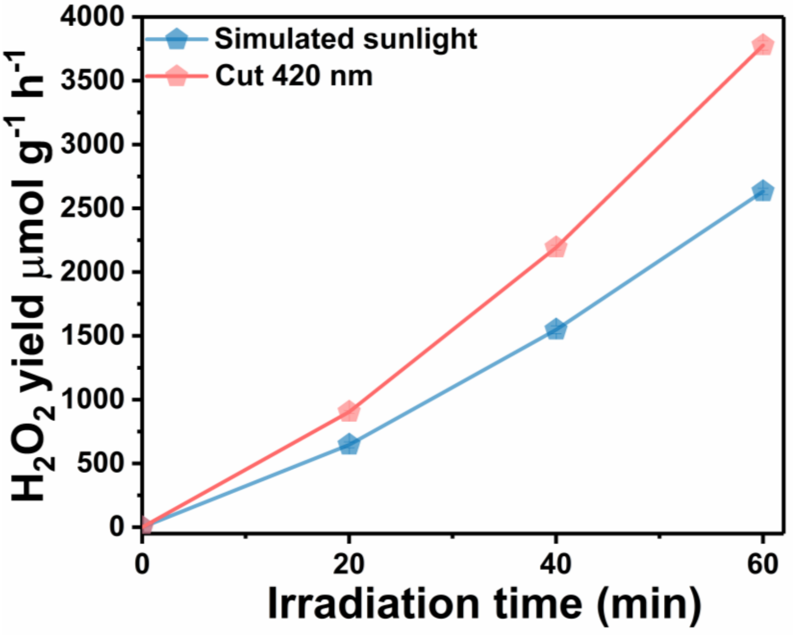

Figure S6 Time-dependent H_2O_2 rate curves of PI-BD-TPB under different light sources.

PI-BD-TPB photocatalyst with high H_2O_2 generation efficiency presented the H_2O_2 production rate of 2632.88 μmol g^{-1} h^{-1} under visible light irradiation.

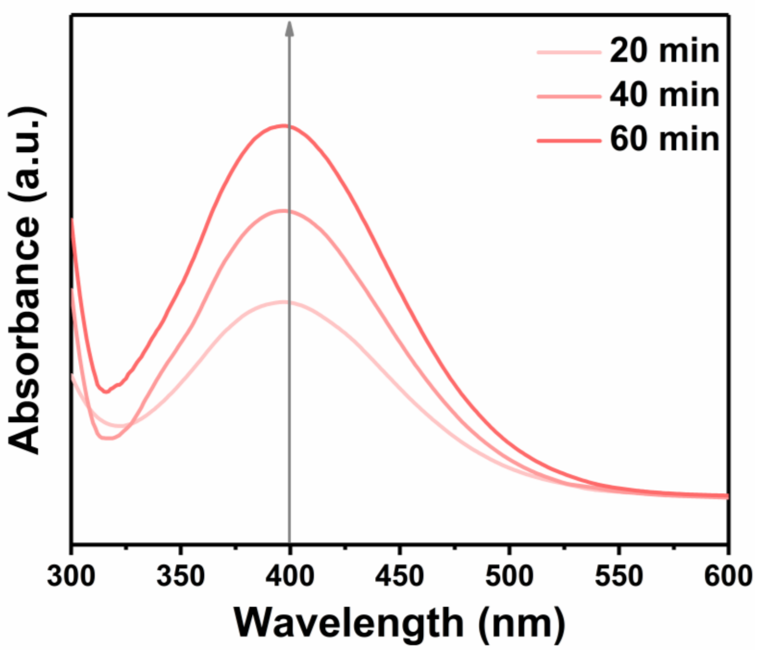

Figure S7 UV-vis absorption spectra of potassium titanium oxalate after reacting with produced H_2O_2 on BD-TPB photocatalyst in 60 min under simulated sunlight.

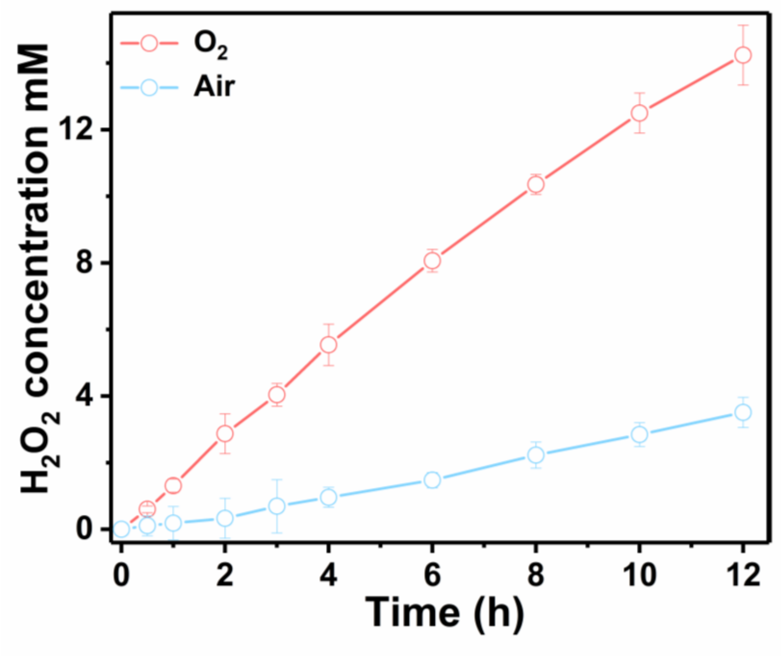

Figure S8 Photocatalytic H_2O_2 accumulation of BD-TPB under simulated sunlight irradiation with O_2 or air atmosphere.

2.4 The stability of BD-TPB photocatalyst

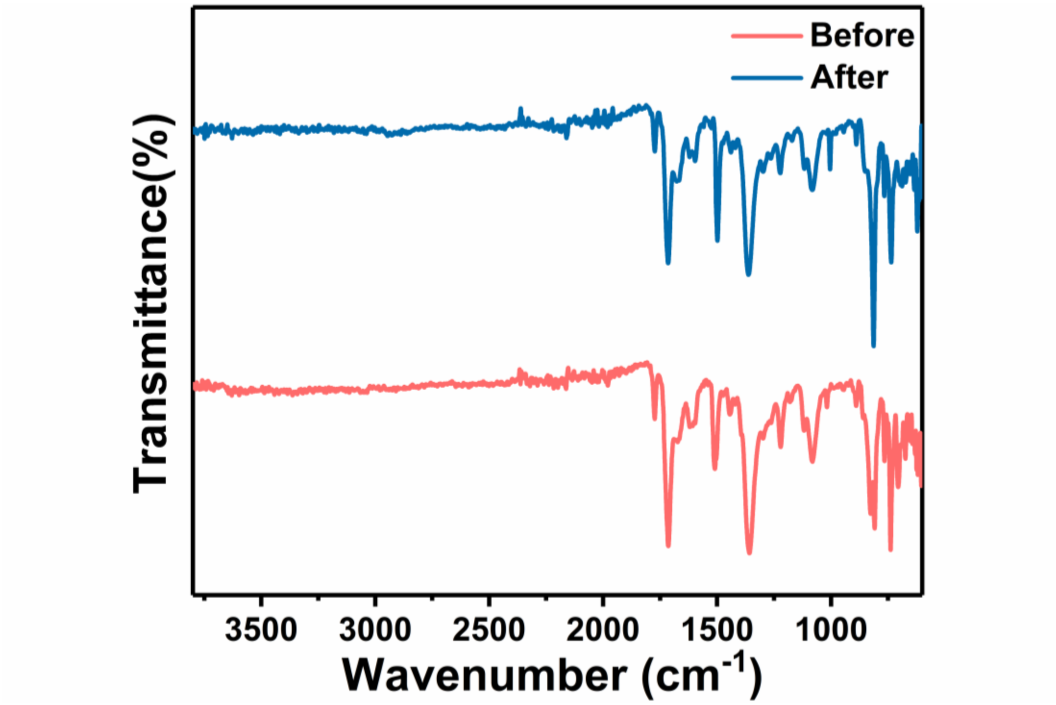

Figure S9 FTIR spectra of BD-TPB photocatalyst after long-term reaction.

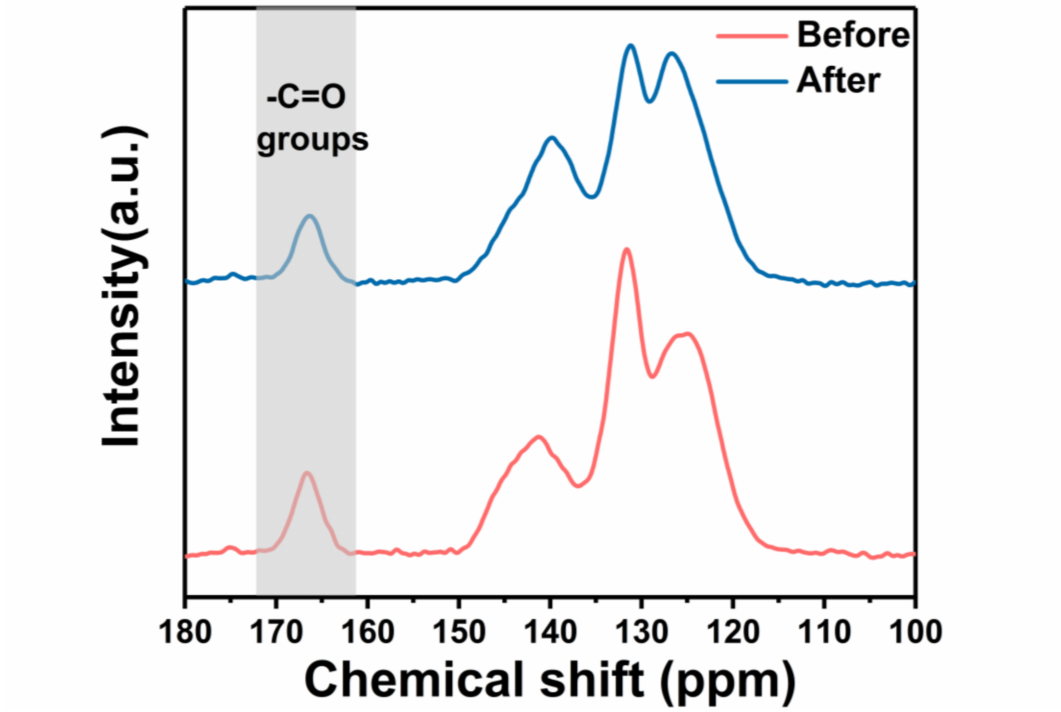

Figure S10 The solid-state ^{13}C NMR spectra of BD-TPB photocatalyst after long-term reaction.

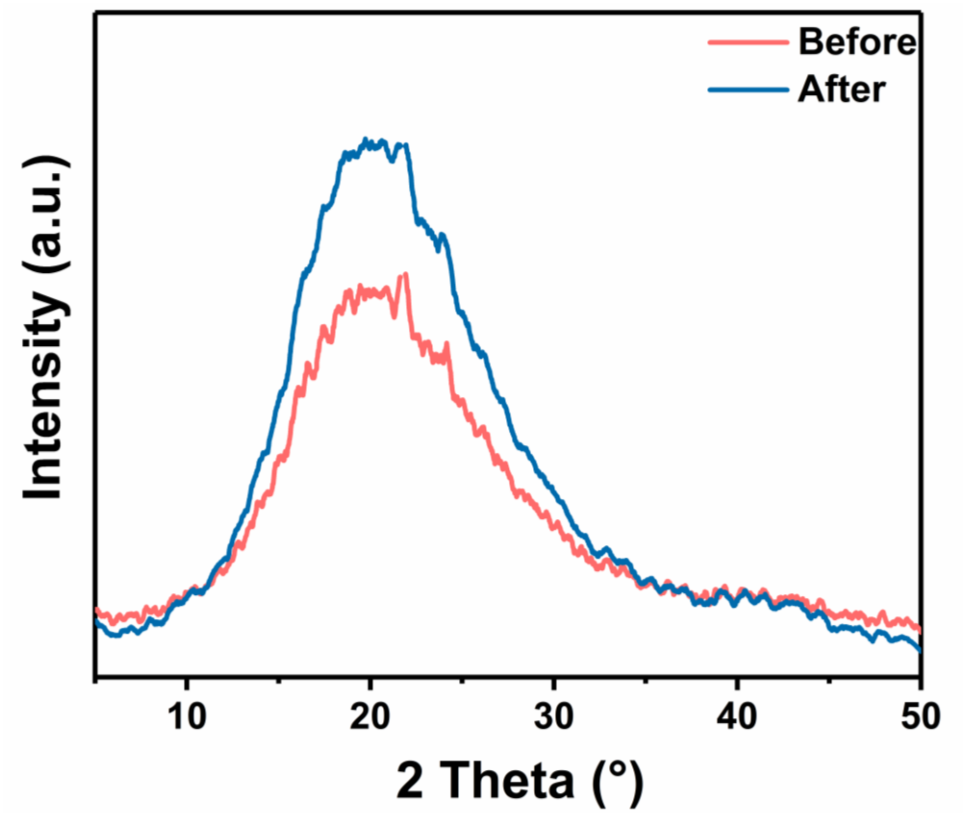

Figure S11 The XRD spectra of BD-TPB photocatalyst after long-term reaction.

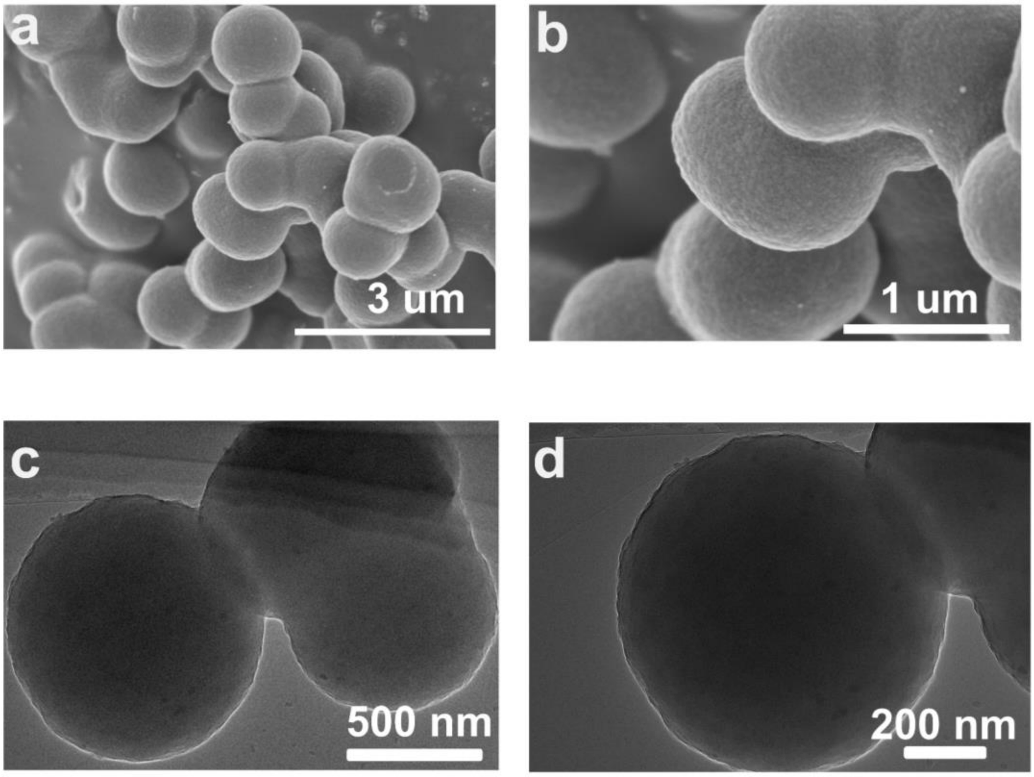

Figure S12 (a) and (b) SEM of BD-TPB photocatalyst after long-term reaction; (c) and (d) TEM of BD-TPB photocatalyst after long-term reaction.

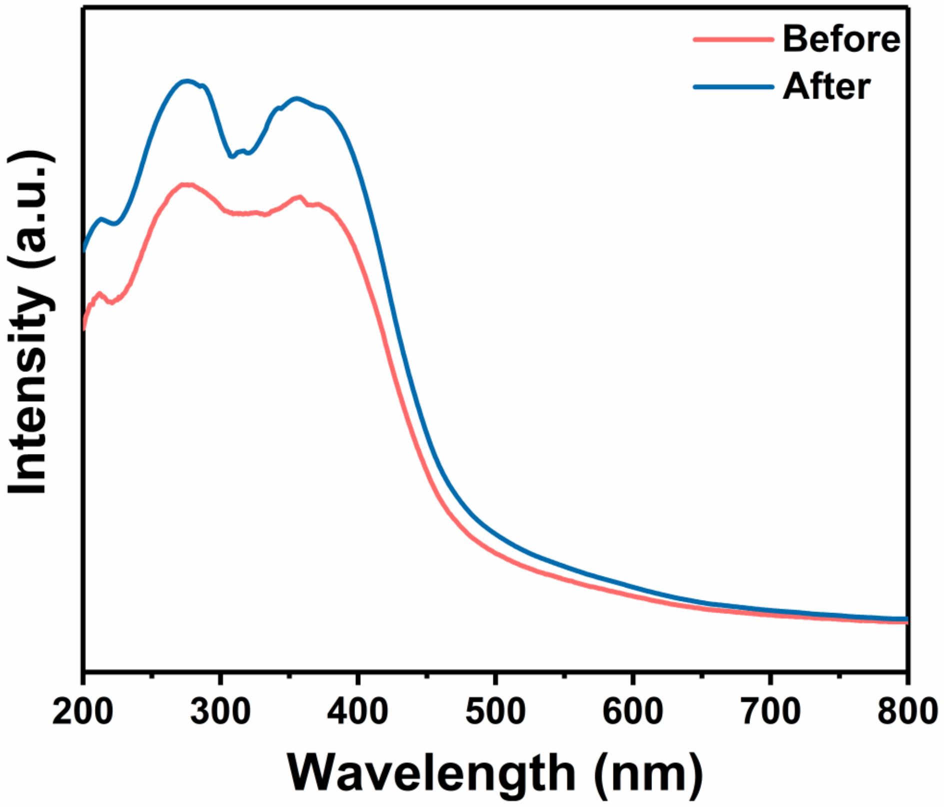

Figure S13 UV-vis diffuse reflectance spectrum for BD-TPB after long-term reaction.

2.5 The SCC of BD-TPB photocatalyst

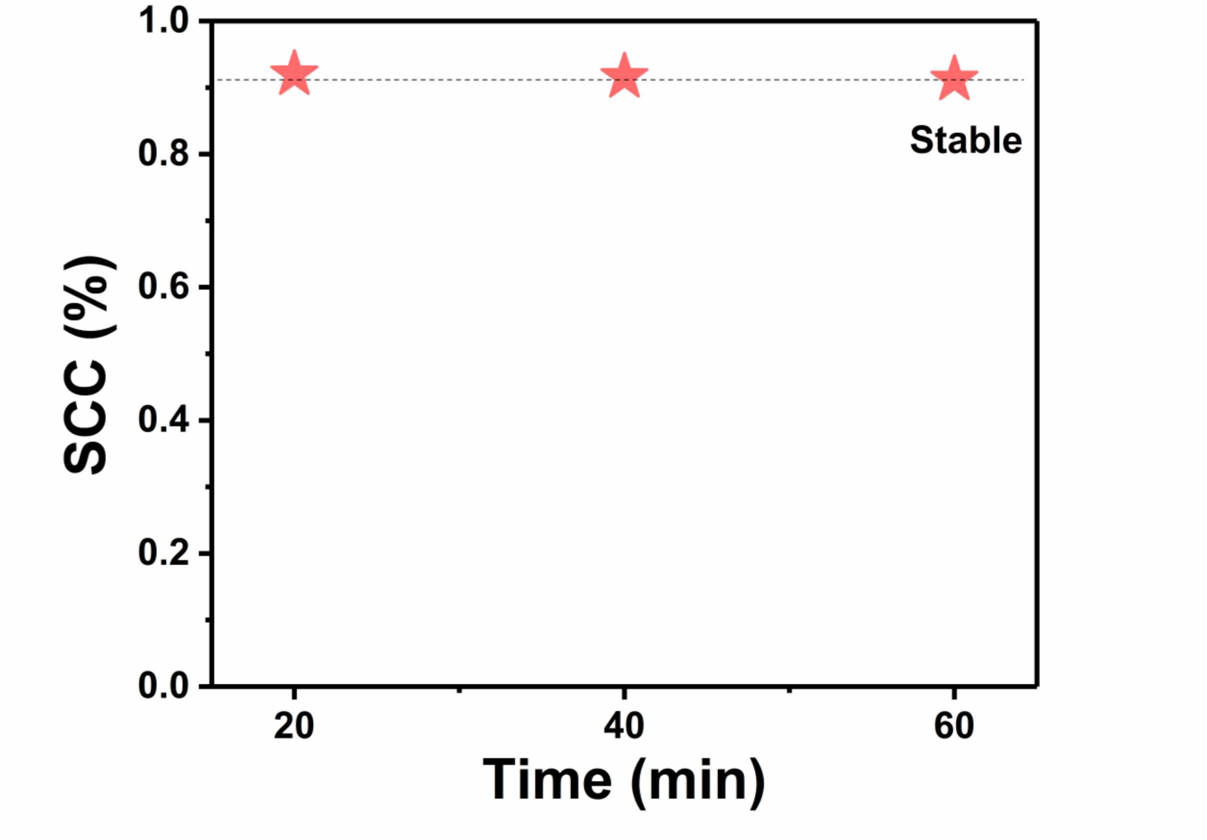

Figure S14 The solar-to-chemical energy conversion (SCC) efficiency of BD-TPB for H_2O_2 production from water and O_2 in 60 min. (Three replicate measurements were averaged).

SCC efficiency was determined by the photocatalytic experiments. 80 mg photocatalysts powder and 60 mL water were added into a flask and bubbled with O_2 for 30 min, the reaction was carried out at 50 °C in water bath. The SCC efficiency was calculated via following equation: where the free energy (ΔG) for H_2O_2 formation is 117 kJ mol^{-1}, the irradiance of the spectrum is 1,000 W m^{-2} and the irradiated area is 0.785×10^{-4} m^2. The total input energy was therefore 0.0785 W. Reaction time is 1200 s. The average H_2O_2 production of BD-TPB was 7.42 μmol.

The SCC was calculated as follows:

$$SCC (\%) = (\Delta G \times H_2O_2 \text{ formed (mol)}) / (I \times A \times t) \times 100\% = 0.92\%$$

2.6 Photocatalytic decomposition of hydrogen peroxide

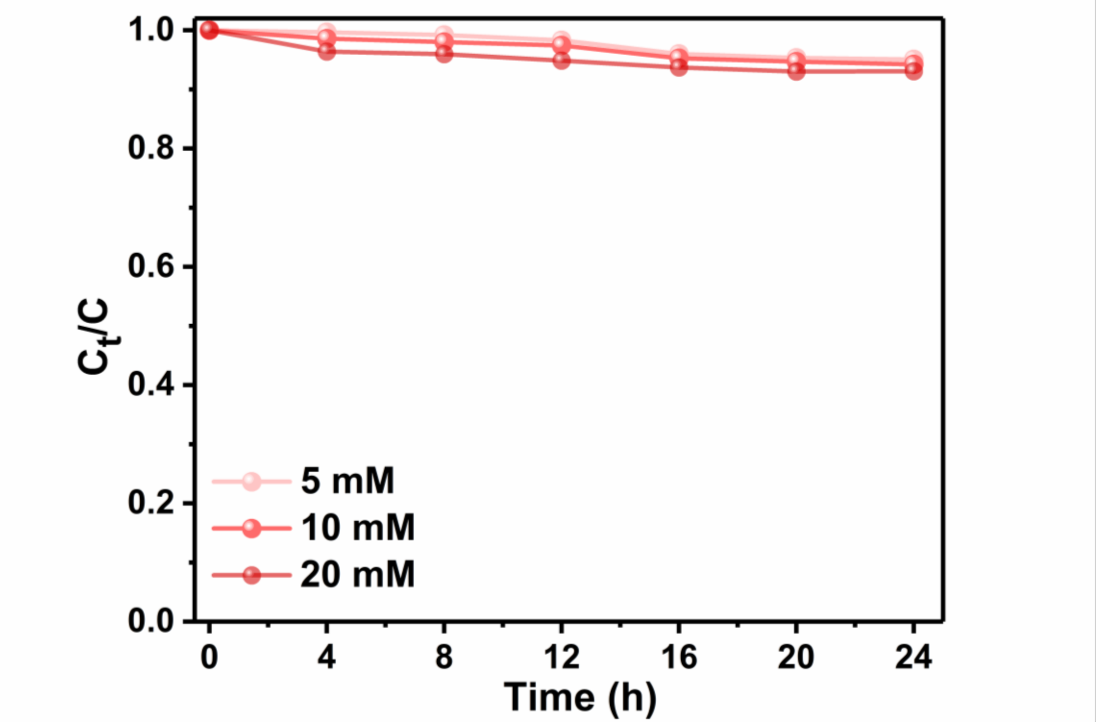

Figure S15 Different H_2O_2 concentrations (C=5 mM, 10 mM and 20 mM) photo-decomposed on BD-TPB photocatalyst under simulated sunlight and Ar atmosphere.

Photocatalytic decomposition of H_2O_2 on BD-TPB: The decomposition of H_2O_2 was conducted by photocatalysts (20 mg) in aqueous solution (20 mL) containing H_2O_2 (5 mM, 10 mM and 20 mM) under Ar atmosphere and simulated sunlight. The concentration of H_2O_2 was measured by potassium titanium oxalate method. The results showed that more than 94% H_2O_2 was maintained after irradiation for 24 h, revealing BD-TPB deficient activity.

2.7 Practical performance tests of BD-TPB photocatalyst

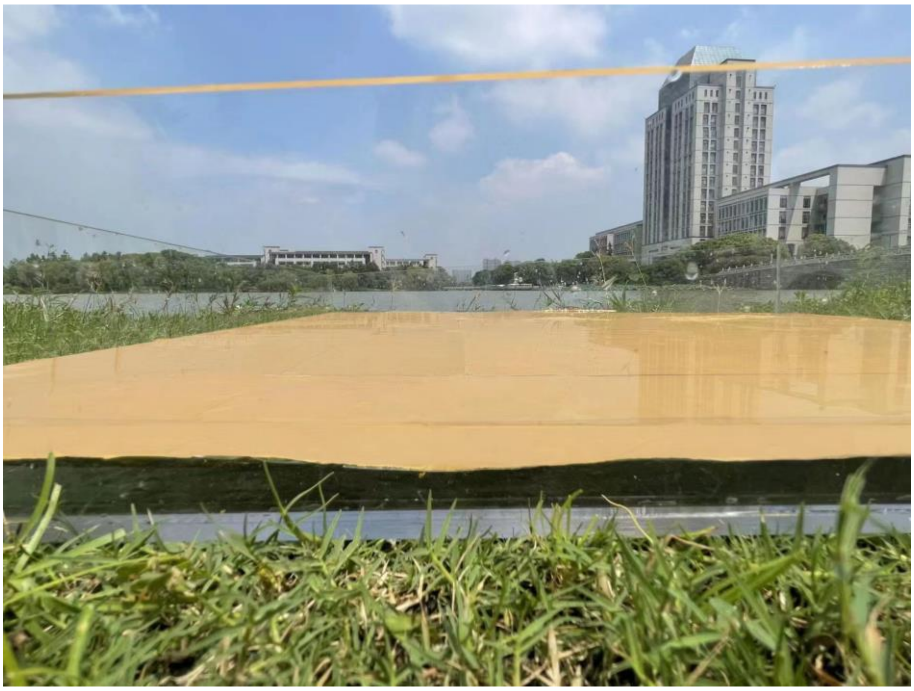

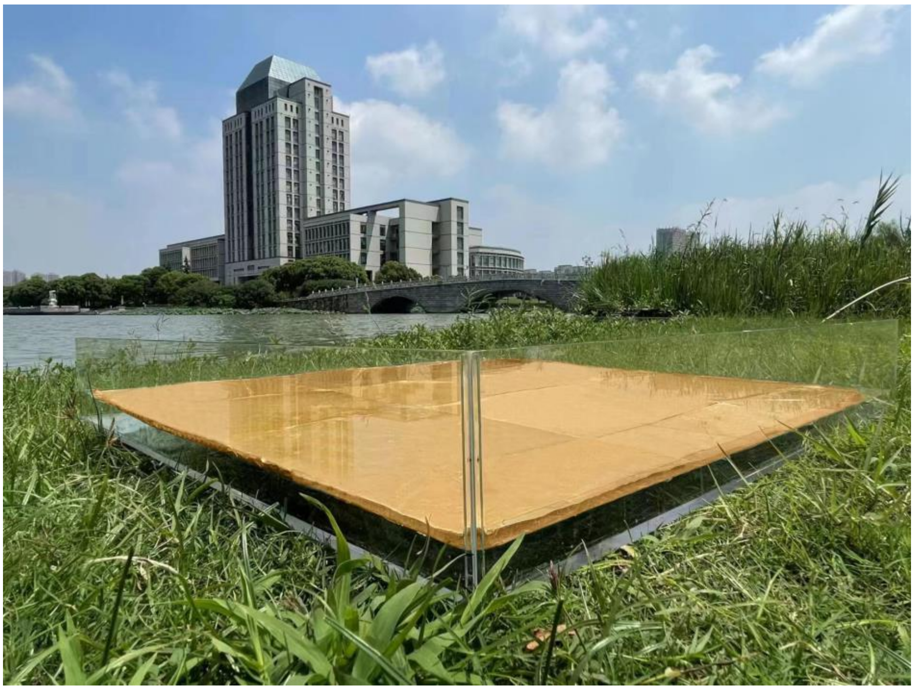

Figure S16 Digital images of the scalable generation of H_2O_2 measurements under natural sunlight on the Jiang Nan University campus using the PI-BD-TPB membrane as the photocatalyst.

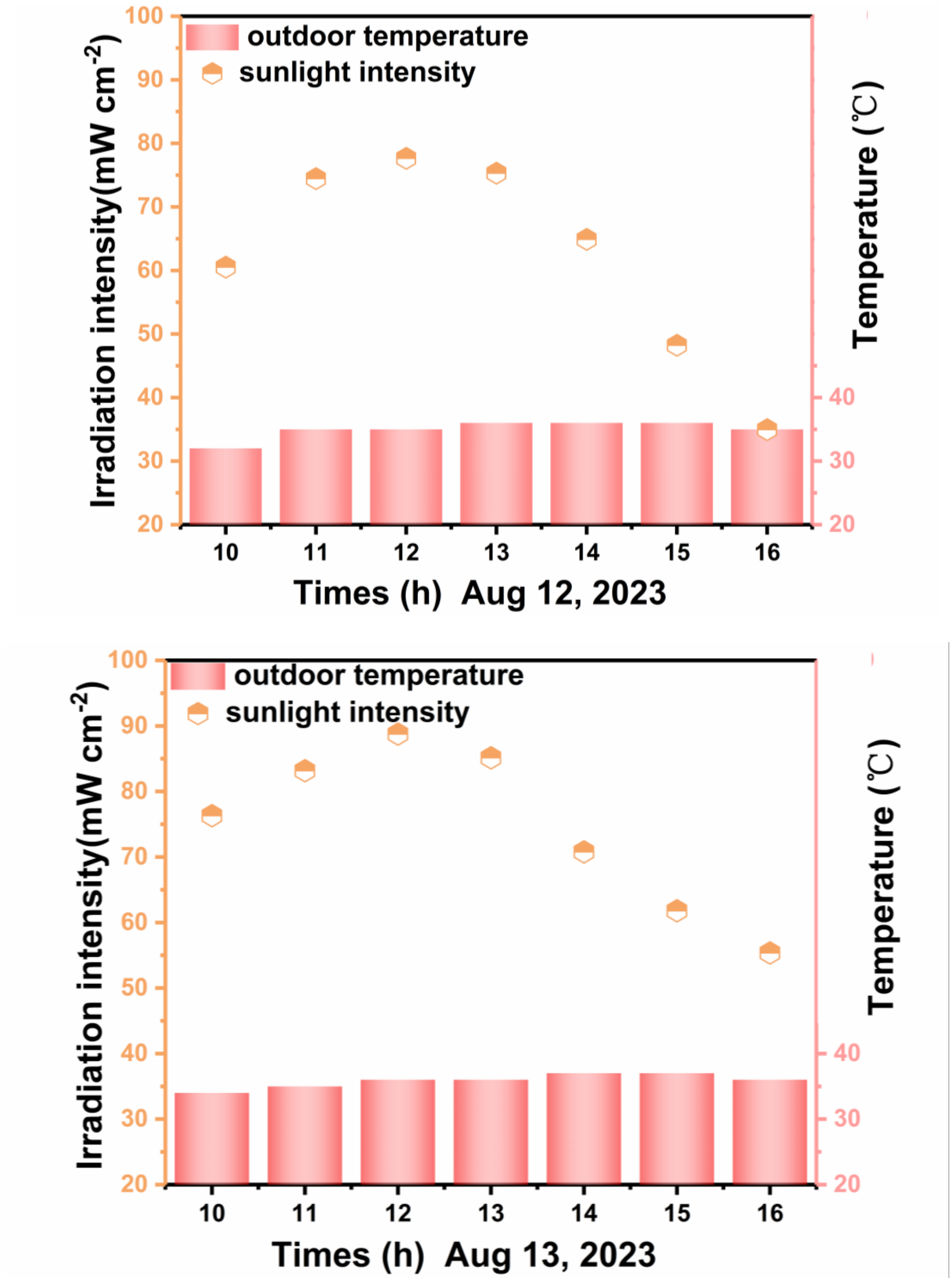

Figure S17 Photosynthesis of H_2O_2 in ambient condition. The intensity of sunlight and temperature at the corresponding time in the outdoor environment of Jiang Nan University campus from 10:00 am to 4:00 pm on Aug 12, 2023; The intensity of sunlight and temperature at the corresponding time in the outdoor environment of Jiang Nan University campus from 10:00 am to 4:00 pm on Aug 13, 2023.

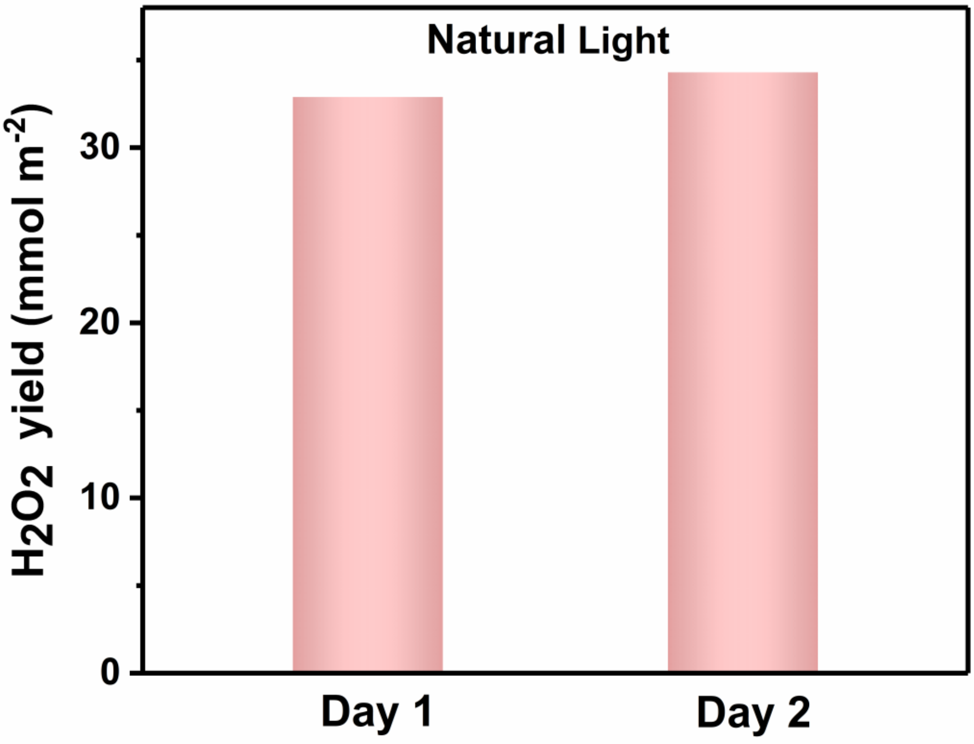

Figure S18 Practical performance tests based on the PI-BD-TPB membrane under natural sunlight (6 h each day).

Photosynthesis of H_2O_2 in ambient conditions: the intensity of sunlight and temperature at the corresponding time in the outdoor environment of Jiang Nan University campus from 10:00 am to 4:00 pm on Aug 12, 2023; the intensity of sunlight and temperature at the corresponding time in the outdoor environment of Jiang Nan University campus from 10:00 am to 4:00 pm on Aug 13, 2023.

2.8 Comparison of photocatalytic H_2O_2 activity

Table S1. Comparison of H_2O_2 production of polymer photocatalyst

**Photocatalyst**	**Catalyst dose**	**Light source**	**H_2O_2 yields**	**Cycle times**	**Year**	**Ref.**
APFac	0.2 g/L	λ≥420 nm	224 μM/h	7 h	2023	2
Bpy-TAPT	0.16 g/L	λ≥420 nm	673 μM/h	24 h	2023	3
TD-COF	0.25 g/L	White LED	1155 μM/h	10 h	2023	4
COF-N32	0.5 g/L	λ≥420 nm	302 μM/h	60 h	2023	5
Tz-COF	0.5 g/L	λ≥420 nm	402 μM/h	270 min	2023	6
C_5N_2	10 g/L	λ≥420 nm	698 μM/h	24 h	2022	7
COF-TfbBpy	0.5 g/L	sun light	1042 μM/h	60 h	2022	8
Bpt-CIF	0.2 g/L	λ≥420 nm	653 μM/h	10 h	2022	9
N_3-COF	0.5 g/L	495 nm LED	785 μM/h	20 h	2022	10
HTCC	0.2 g/L	λ≥400 nm	232 μM/h	4 h	2021	11
TPE-AQ	0.25 g/L	AM 1.5	227 μM/h	20 h	2021	12
TPB-DMTP	0.2 g/L	λ≥420 nm	580 μM/h	300 min	2021	13
DE7	1.6 g/L	λ≥420 nm	333 μM/h	96 h	2021	14
CHF-DPDA	2.0 g/L	λ≥420 nm	3450 μM/h	13.5 h	2021	15
RF/P3HT-1.0	1.6 g/L	λ≥420 nm	185 μM/h	50 h	2021	16
PEI/C_3N_4	1.0 g/L	AM 1.5	208 μM/h	180 min	2020	17
Sb-SAPC15	2.0 g/L	λ≥420 nm	12.4 mg l^{-1}	20 h	2020	18
RF-base resins	1.6 g/L	λ≥420 nm	1240 μM/h	10 h	2019	19
CTF-BDDBN	2.0 g/L	AM 1.5	57.5 μM/h	72 h	2019	20
C_3N_4/PDIBN.rGO	1.0 g/L	λ≥420 nm	530 μM/h	24 h	2018	21
C_3N_4/MTI	1.6 g/L	λ≥420 nm	42 μM/h	60 h	2017	22
C_3N_4/BDI	1.6.g/L	λ≥420 nm	16 μM/h	24 h	2016	23
**PI-BD-TPB**	**1.5 g/L**	**AM 1.5**	**2850 μM/h**	**144 h**	**2024**	

Section 3. Characterizations of PI-BD-TPB photocatalyst

3.1 XPS spectra of BD-TPB

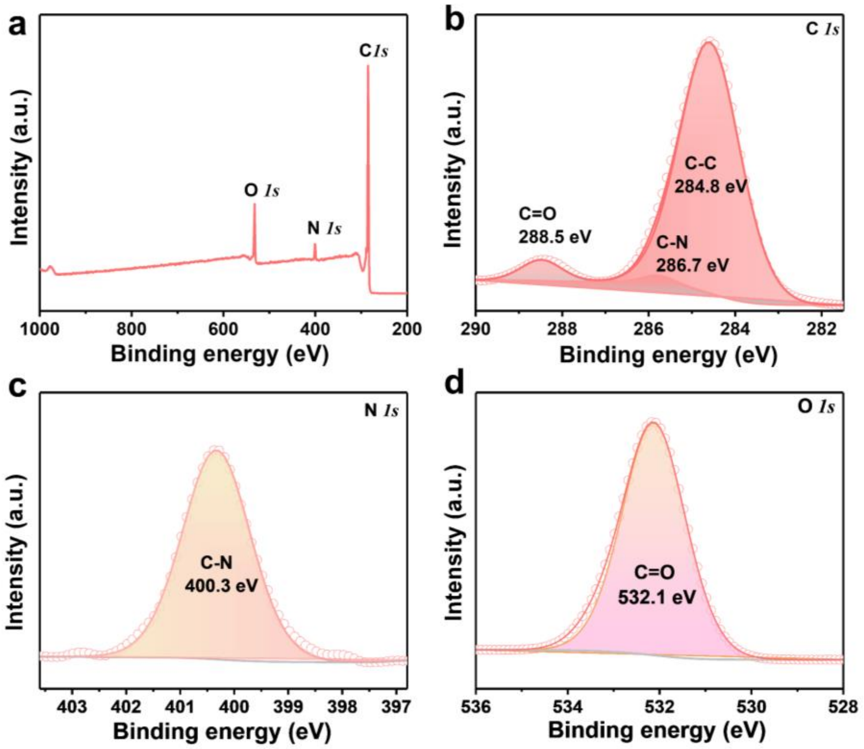

Figure S19 XPS survey for BD-TPB (a) XPS survey spectra, (b) C *1s*, (c) N *1s* and (d) O *1s*.

The XPS survey spectrum showed the C, N and O elements for PI-BD-TPB. The high-resolution spectra of C *1s* could be deconvoluted to three peaks with binding energies of 284.8 286.7, and 288.5 eV, which could be assigned to C−C/C=C, C−N and C=O, respectively. In the high-resolution spectra of N *1s*, 400.3 eV peak assigned to C=N were observed. For the high-resolution spectra of O *1s*, a typical peak centered at 532.1 eV could be ascribed to C=O.

3.2 GPC spectra of PAA

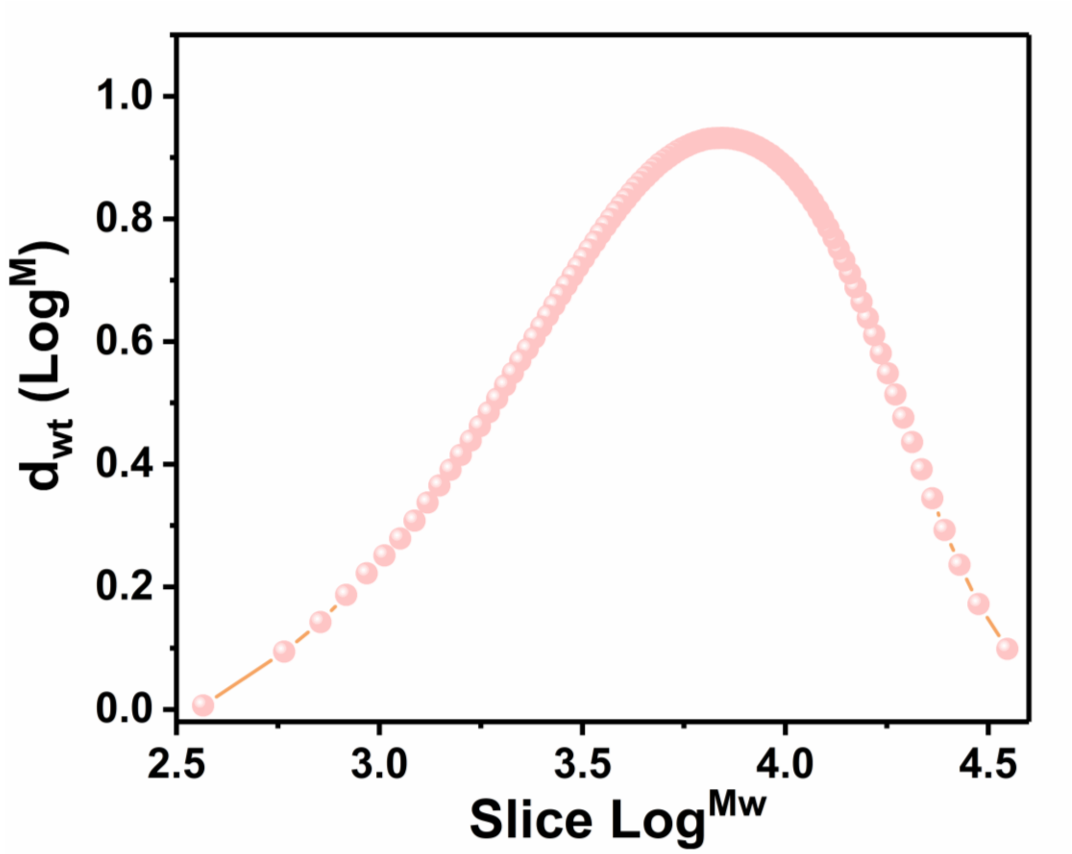

Figure S20 The molecular weight distribution curve via gel permeation chromatography (GPC) of PAA powers. [24]

3.3 SEM analysis of BD-TPB

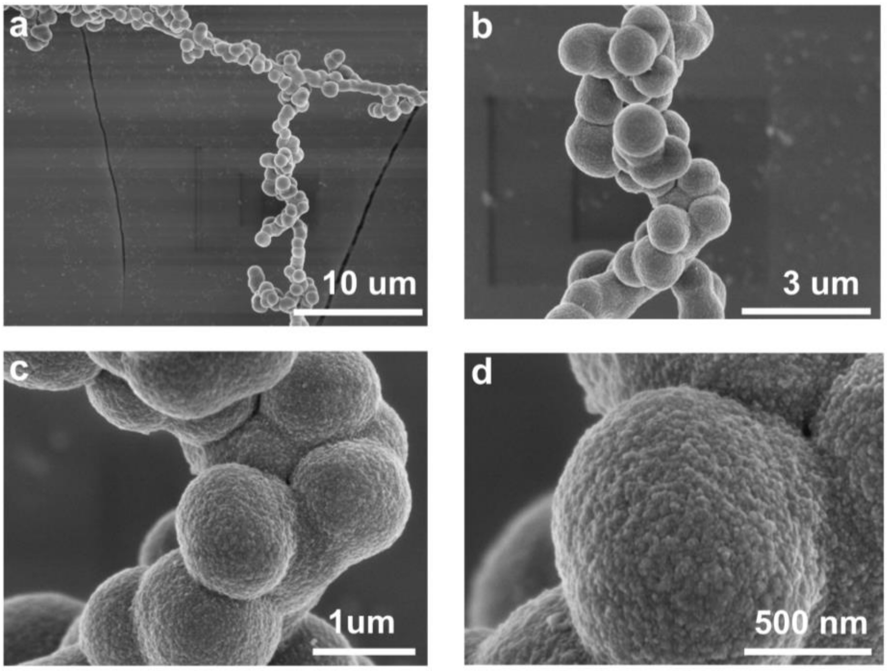

Figure S21 Morphology of BD-TPB aerogel: Scanning electron microscopic (SEM) images at scale bar of (a) 10 μm, (b) 3 μm, (c) 1 μm and (d) 500 nm.

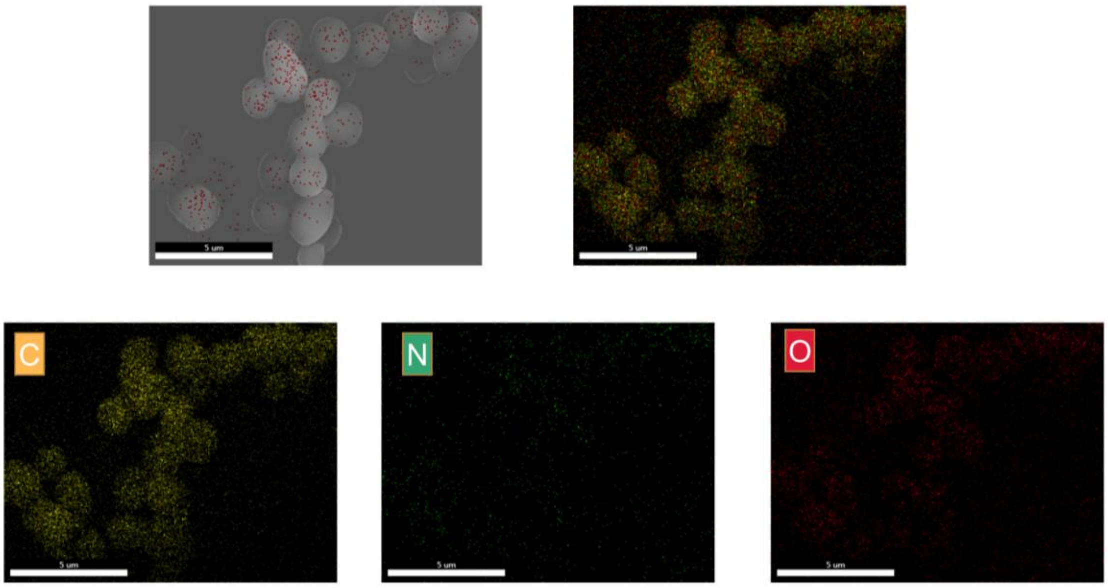

Figure S22 The energy-dispersive X-ray spectroscopy (EDS) mapping images of BD-TPB.

3.4 TEM analysis of BD-TPB

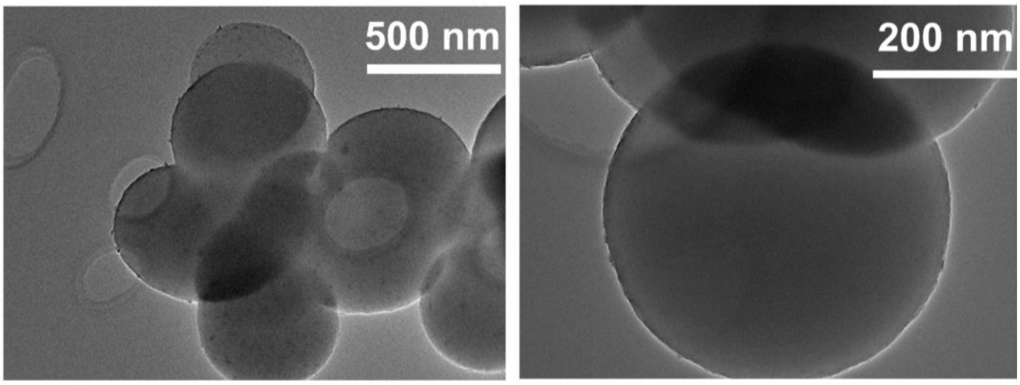

Figure S23 TEM images of BD-TPB aerogel.

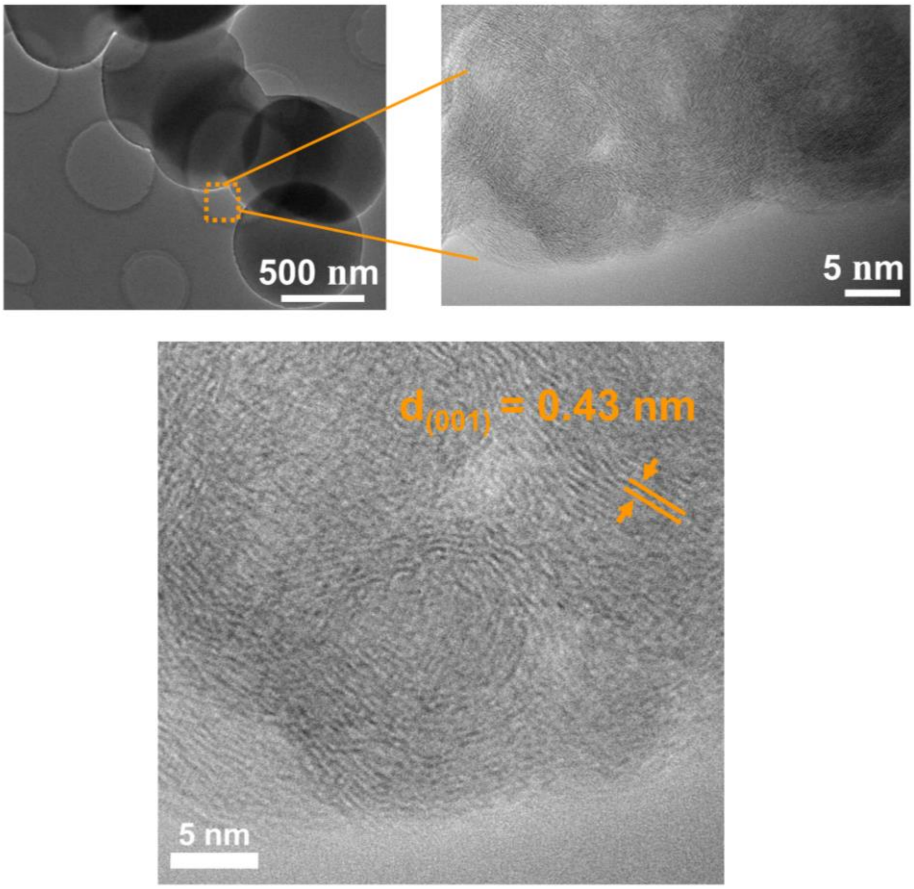

Figure S24 High-resolution transmission electron microscope (HRTEM) images of BD-TPB.

Scanning electron microscopy (SEM) and Transmission electron microscopy (TEM) observation of PI-BD-TPB aerogel showed cross-linked spherical particles with an average diameter of ~1-2 μm (**Figure S21** and **23**). X-ray spectroscopy (EDS) mapping, further confirming the existence of C, N and O element (**Figure S22**).

3.5 XRD analysis of BD-TPB

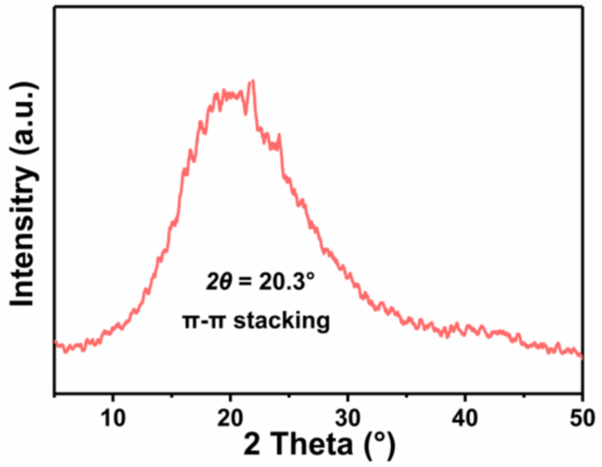

Figure S25 The XRD spectra of BD-TPB aerogel.

3.6 Nitrogen isotherm and pore size distribution of BD-TPB

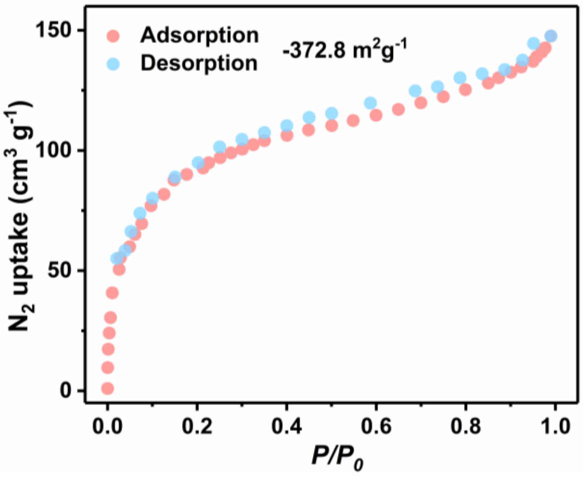

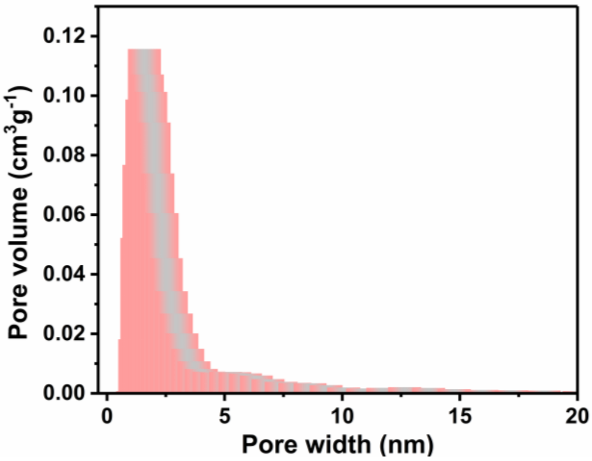

Figure S26 Nitrogen adsorption and desorption isotherm of the BD-TPB aerogel and pore size distribution.

3.7 Mercury intrusion measurement of BD-TPB

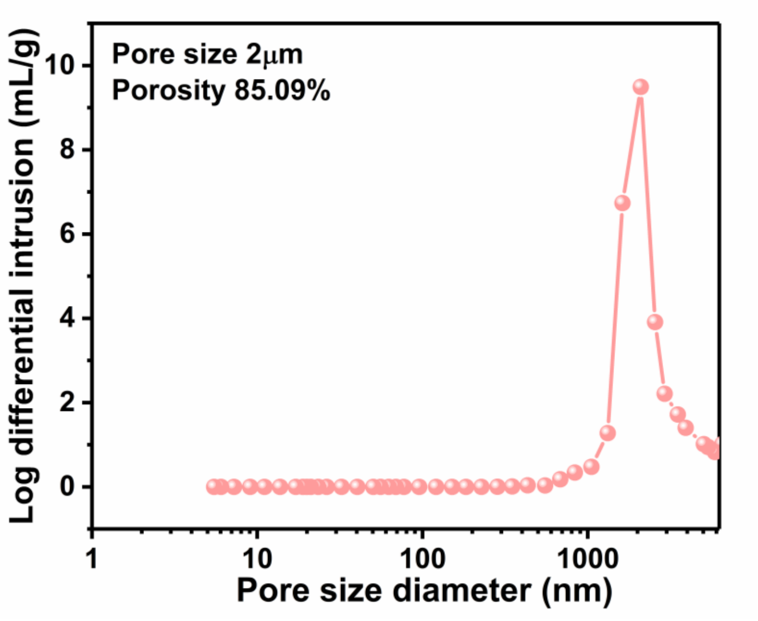

Figure S27 Pore size distribution of the BD-TPB based on mercury intrusion porosimetry. [25]

3.8 TGA analysis of BD-TPB

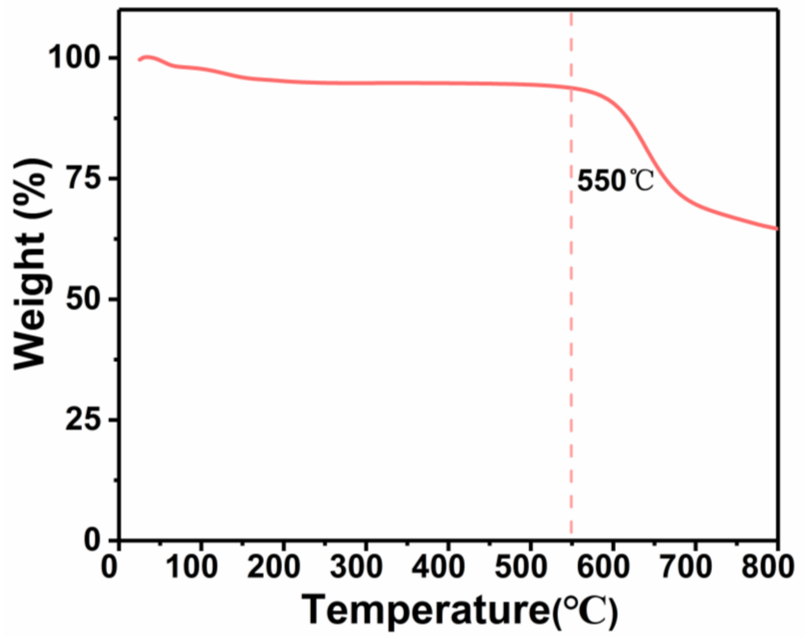

Figure S28 Thermal gravimetric analysis curve of the BD-TPB.

3.9 Chemical stability

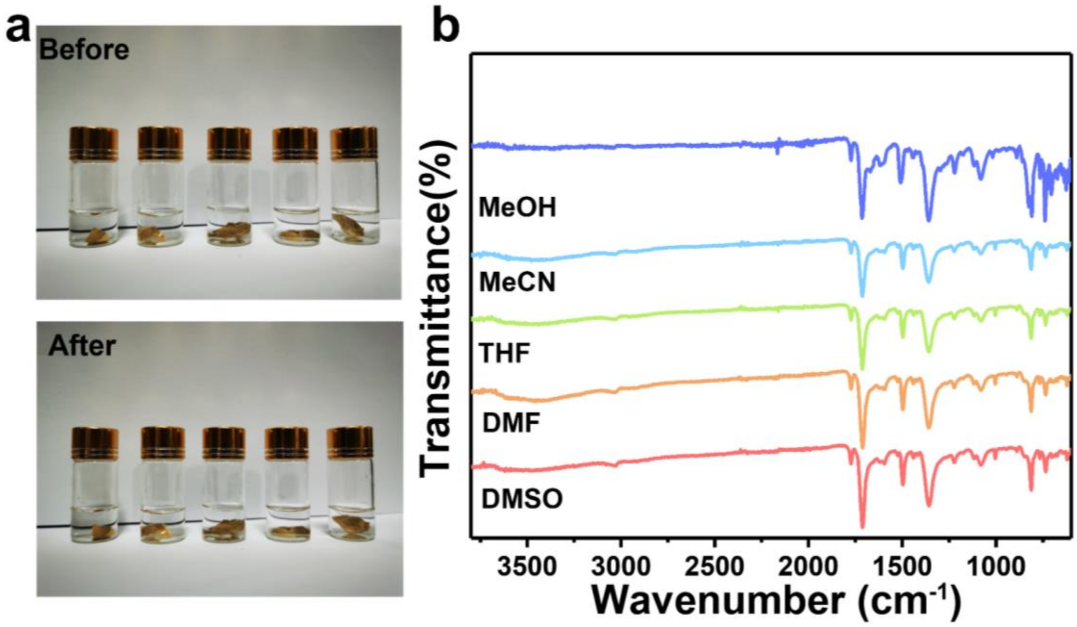

Figure S29 Stability testing for macroscopic BD-TPB aerogel immersed in MeOH, MeCN, THF, DMF and DMSO, respectively, and the treated FTIR spectra, (a) and (b) of BD-TPB.

The BD-TPB aerogel was soaked in MeOH, MeCN, THF, DMF and DMSO for 7 days at room temperature, then separated by centrifugation, washed three times with 1-methayl-2-pyrrolidinone containing ethanol to remove the other impurities and freeze-dried. BD-TPB aerogel was then performed for FTIR measurement. As shown **Figure S29a**, BD-TPB aerogel was stable in MeOH, MeCN, THF, DMF and DMSO. It is noteworthy that FTIR analyses of PI-BD-TPB after soaking in different solvents were almost unchanged, confirming the excellent chemical stability (**Figure S29b**). The outstanding chemical and thermal stability are attributed to the robust imide linkage and highly conjugated structure.

3.10 Zeta potential analysis

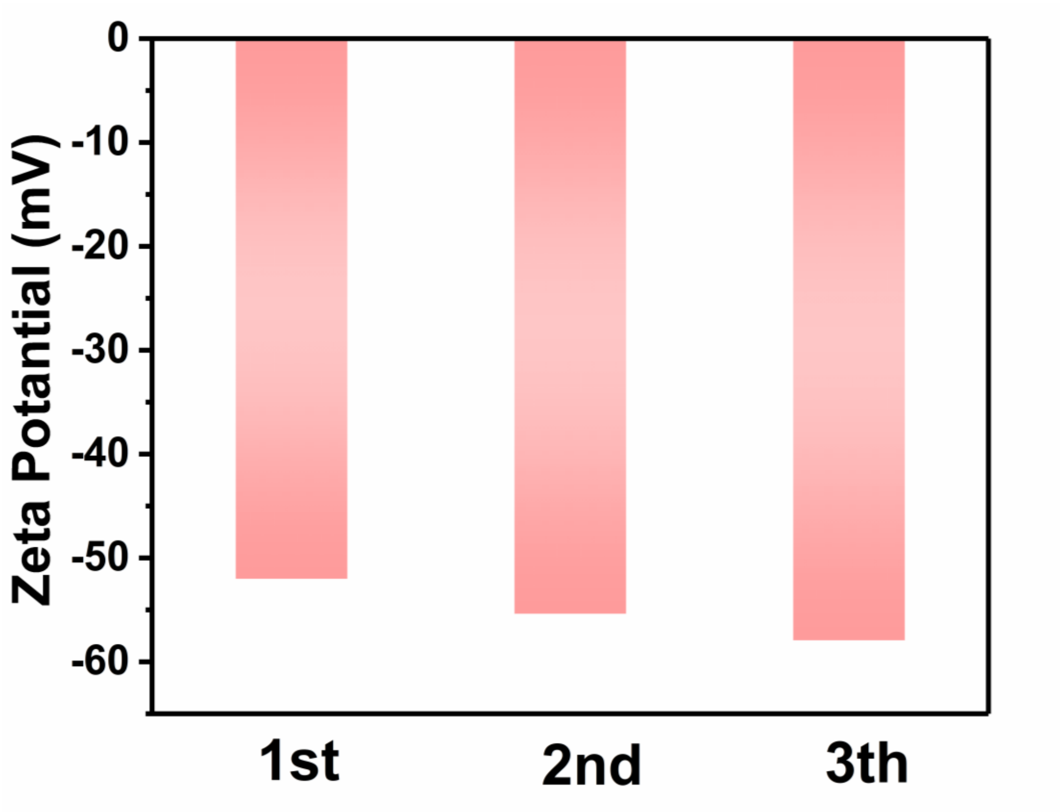

Figure S30 Zeta potential of the BD-TPB in H_2O solution.

3.11 Contact angle measurement of BD-TPB

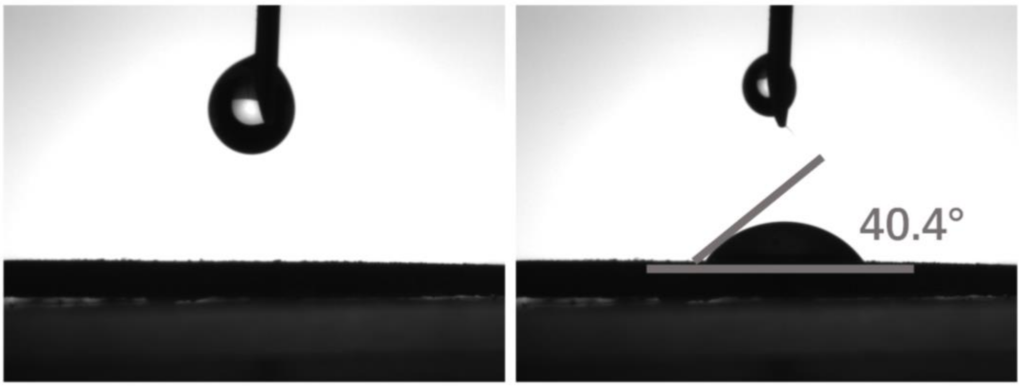

Figure S31 Contact angle measurement of the BD-TPB.

3.12 Additional Characterizations of BD-TPB

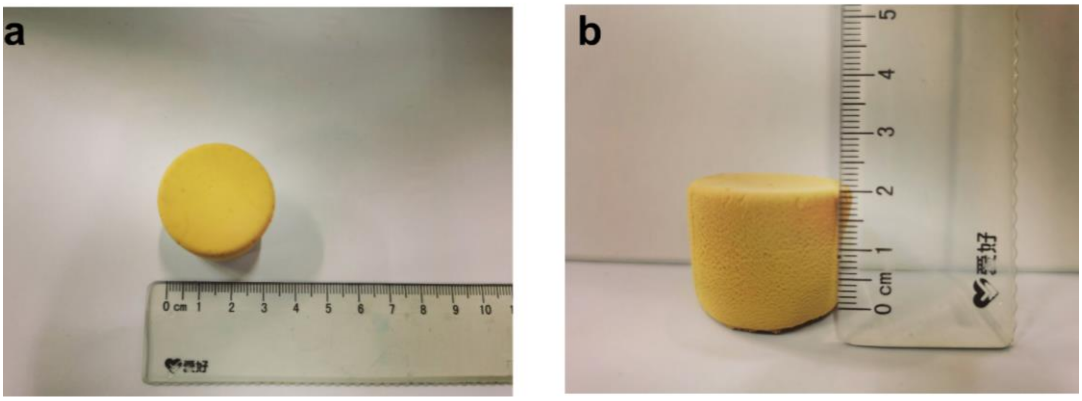

Figure S32 (a) Photograph of BD-TPB aerogel with a diameter of 3 cm; (b) Photograph of BD-TPB aerogel with a height of 2 cm.

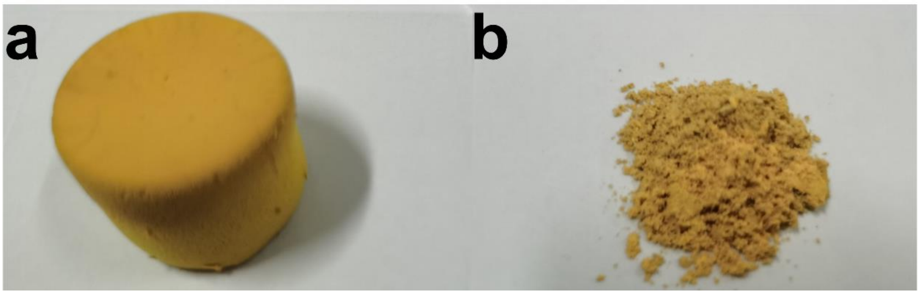

Figure S33 (a) Photographs of polyimide BD-TPB aerogel; (b) Photographs of powder with the same weight.

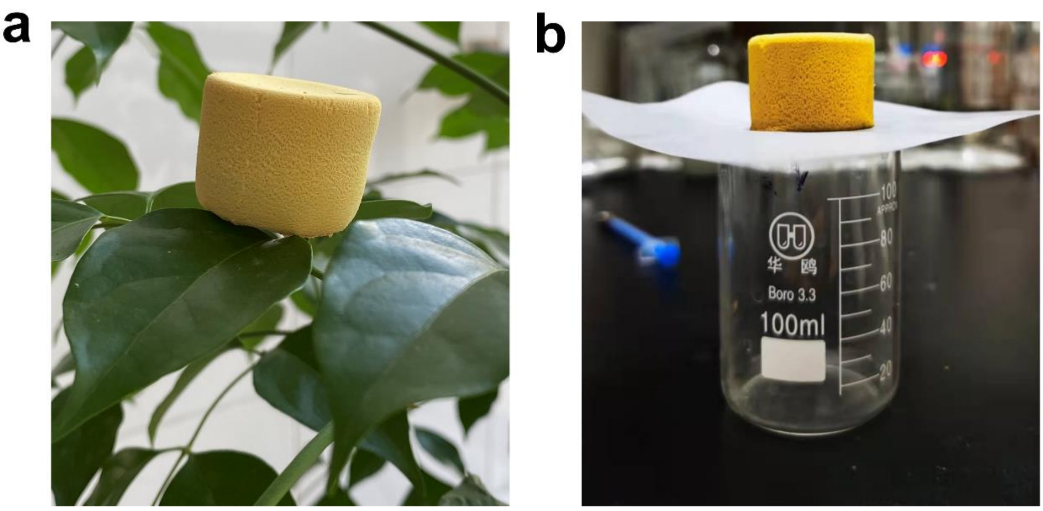

Figure S34 (a) Photograph of the BD-TPB aerogel standing steady on the hairs of leaves ; (b) Photograph of the BD-TPB aerogel standing steady on the hairs of leaves on paper.

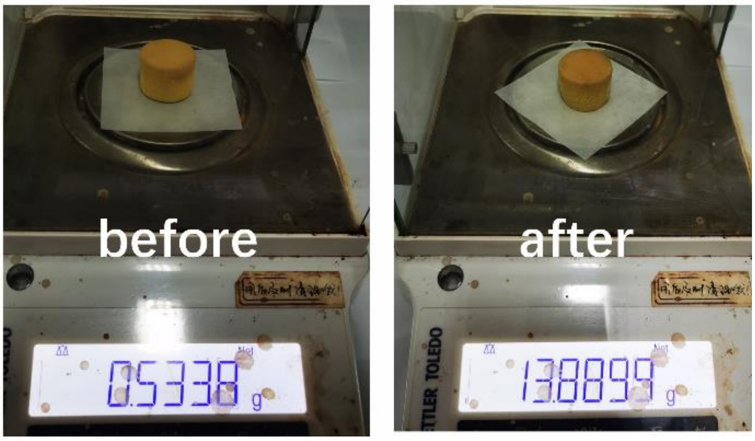

Figure S35 Photographs of the BD-TPB aerogel weighing and absorption capacity for H_2O.

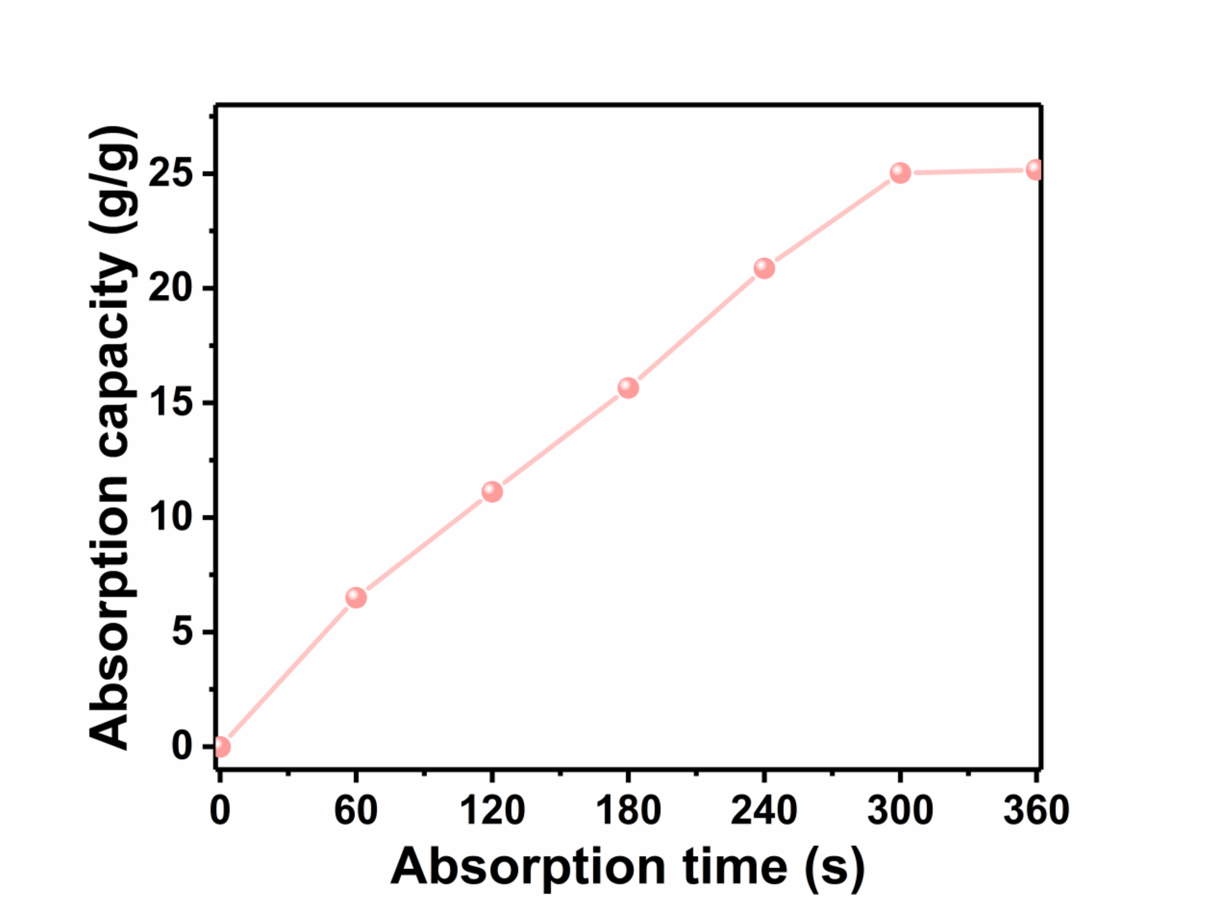

Figure S36 The curve of H_2O absorption capacity of the BD-TPB aerogel.

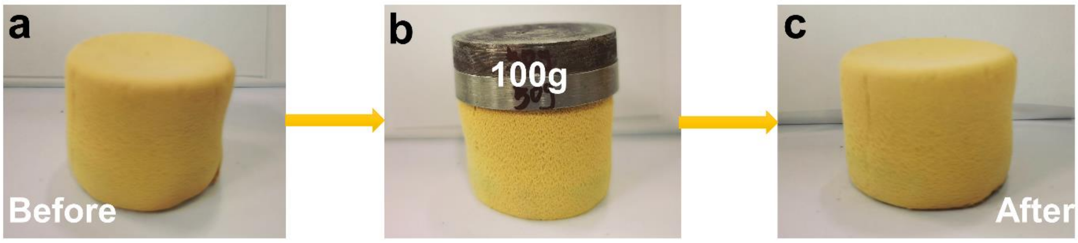

Figure S37 (a) Photo of the BD-TPB aerogel before 100g load; (b) Photo of the BD-TPB aerogel with 100g load and (c) Photo of the BD-TPB aerogel after 100g load.

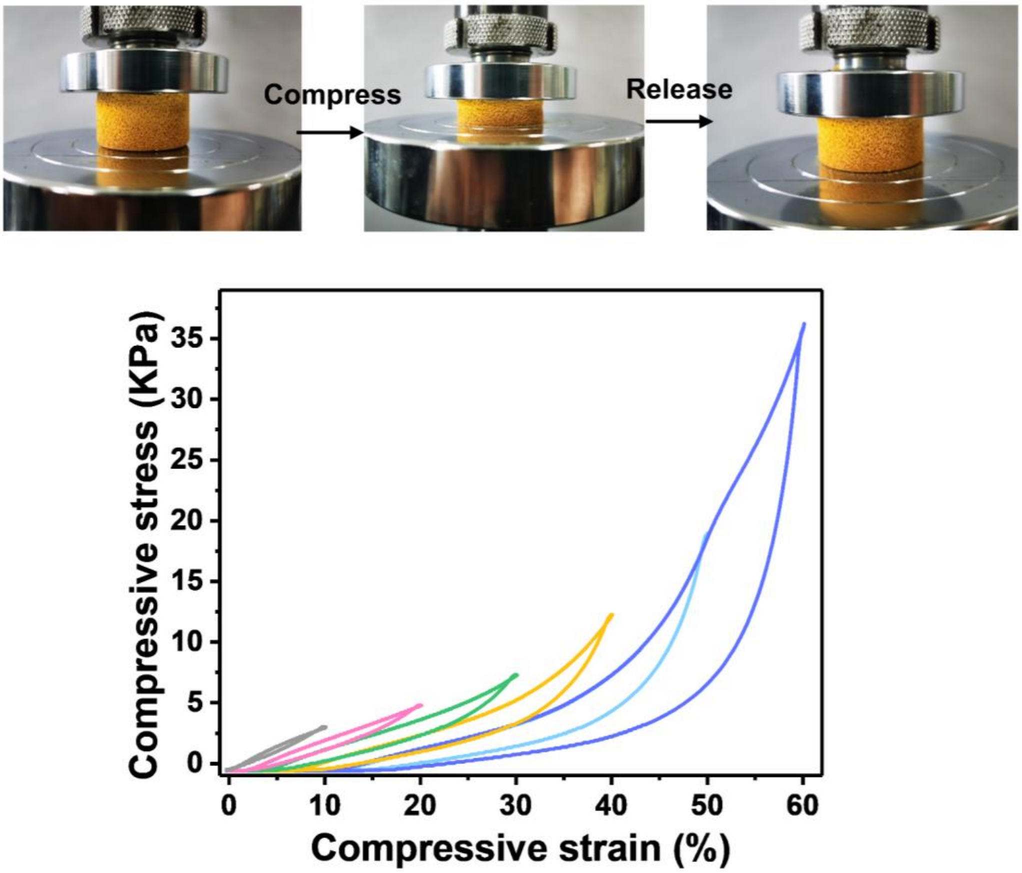

Figure S38 Compressive stress-strain (δ-ε) curves of the BD-TPB at strains of 10%, 20%, 30%, 40%, 50% and 60% and corresponding photographs of the BD-TPB aerogel.

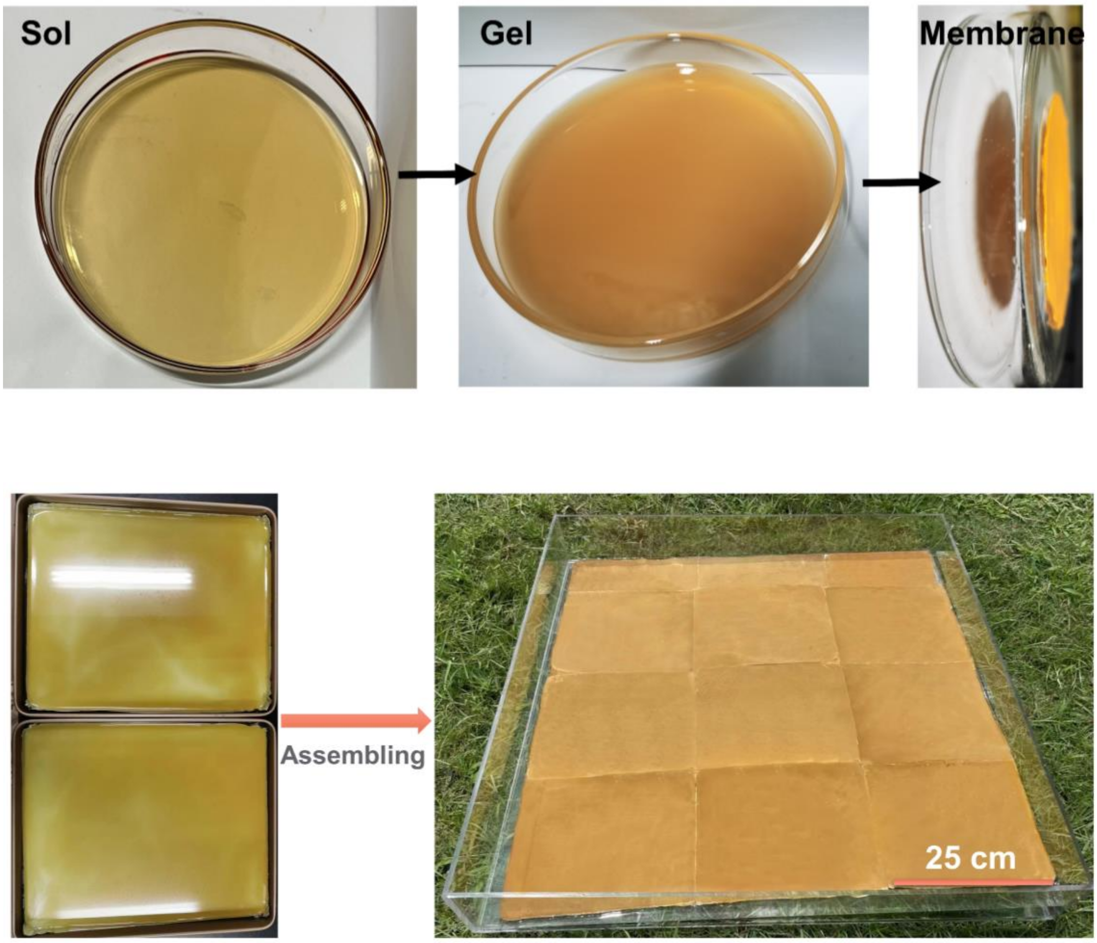

Figure S39 Photographs of the scalable synthesis route of the BD-TPB aerogel membrane and scale-up preparation for practical application.

Section 4. PI-BD-TPB aerogel with reductive carbonyl groups

4.1 Theoretical simulations

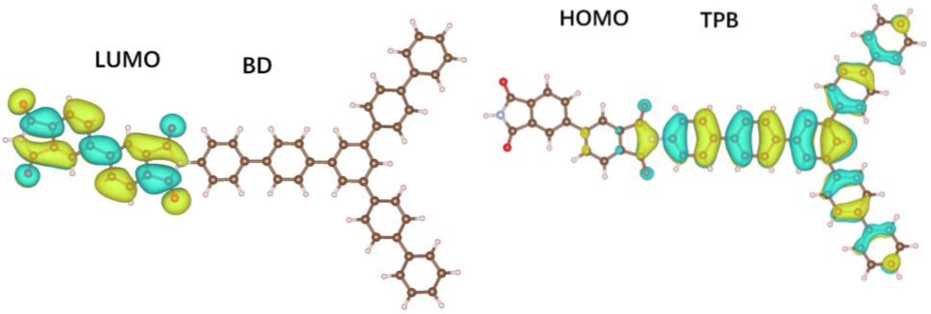

Figure S40 Calculated HOMO-LUMO distribution of BD-TPB unit in the ground state.

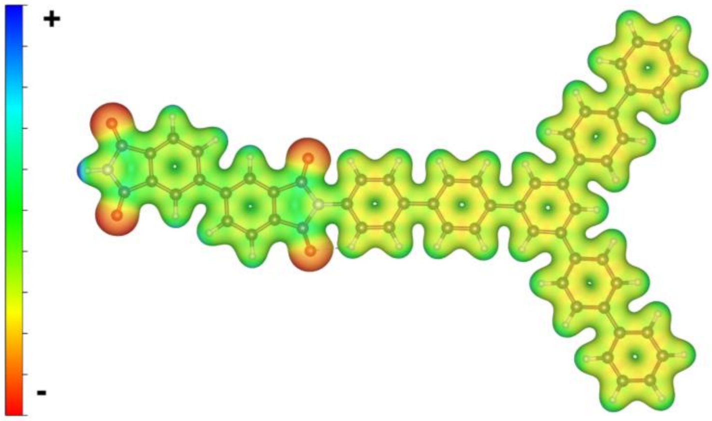

Figure S41 The electrostatic potential distribution of BD-TPB unit in the ground state.

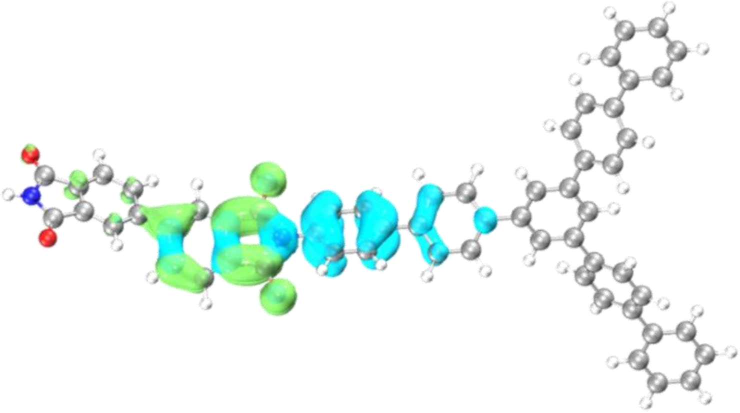

Figure S42 Calculated electron distribution of BD-TPB unit in the excited state.

4.2 Photoelectrochemical measurements

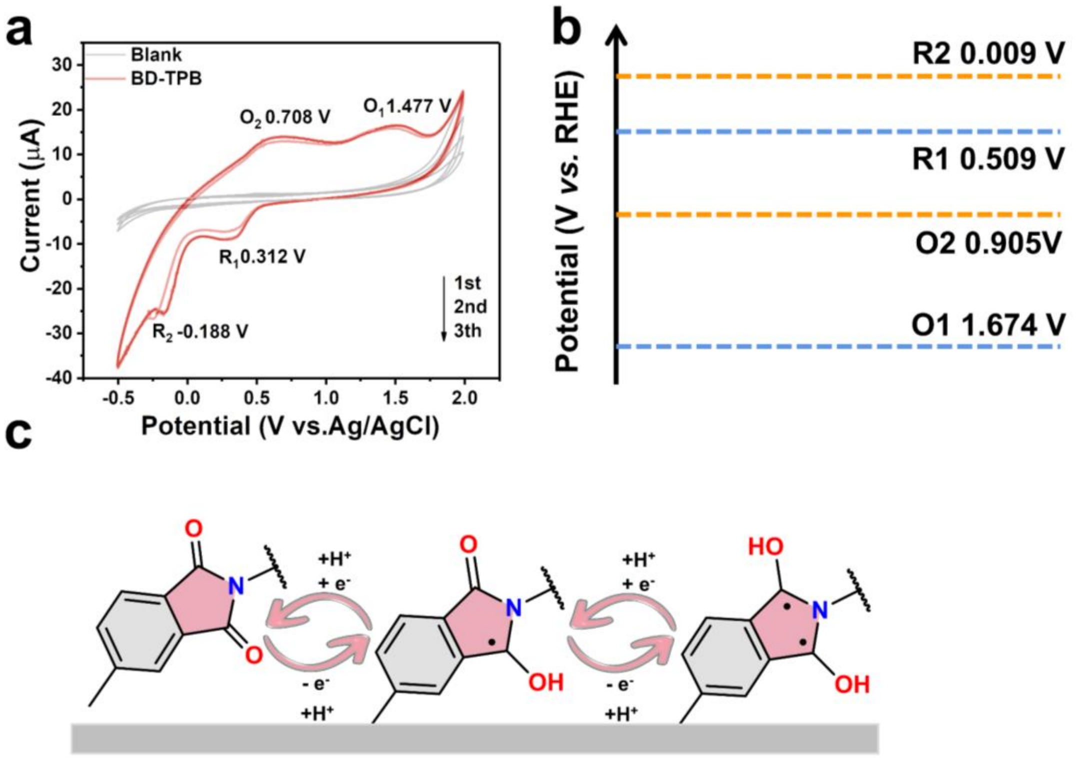

Figure S43 (a) The cyclic voltammetry curve of the PI-BD-TPB electrode at a scan rate of 5 mV s^{-1} in acidic electrolyte solution (0.5 M H_2SO_4) in the dark with three-electrode system; (b) and (c) Schematic illustration of carbonyl groups redox mechanism of the PI-BD-TPB.

The PI-BD-TPB constructed a uniform film on the surface of glassy carbon electrode as a working electrode. Electrode system in which a platinum wire and Ag/AgCl electrode were used as counter electrode and reference electrode, respectively. The cyclic voltammetry (CV) measurements are conducted in the voltage range of -0.5-2.0 V *vs*. Ag/AgCl to investigate the electrochemical properties of PI-BD-TPB in 0.5 M H_2SO_4 electrolyte at a scan rate of 5 mV s^{-1}. As shown in **Figure S43 (a)**, where the two pairs of redox peaks located at 0.312 V / 1.477 V and -0.188 V / 0.708 V (*vs*. Ag/AgCl) are clearly observed, showing the reversible conversion between R-C=O and R-C-OH anion radical. According to the formula E (V *vs*. RHE) = E (V *vs*. Ag / AgCl) + 0.197 V + 0.0591 pH, the reduction potential for BD-TPB is calculated at 0.509 and 0.009 V *vs*. RHE, respectively. Therefore, PI-BD-TPB (E_{CB} = -0.19 V *vs*. RHE) permits the transfer of photogenerated electrons to reduce C=O to C-OH anion (0.509, 0.009 V *vs* RHE). The reduction of R-C=O and to R-C-OH anion radical was to occur during photocatalytic process on PI-BD-TPB.

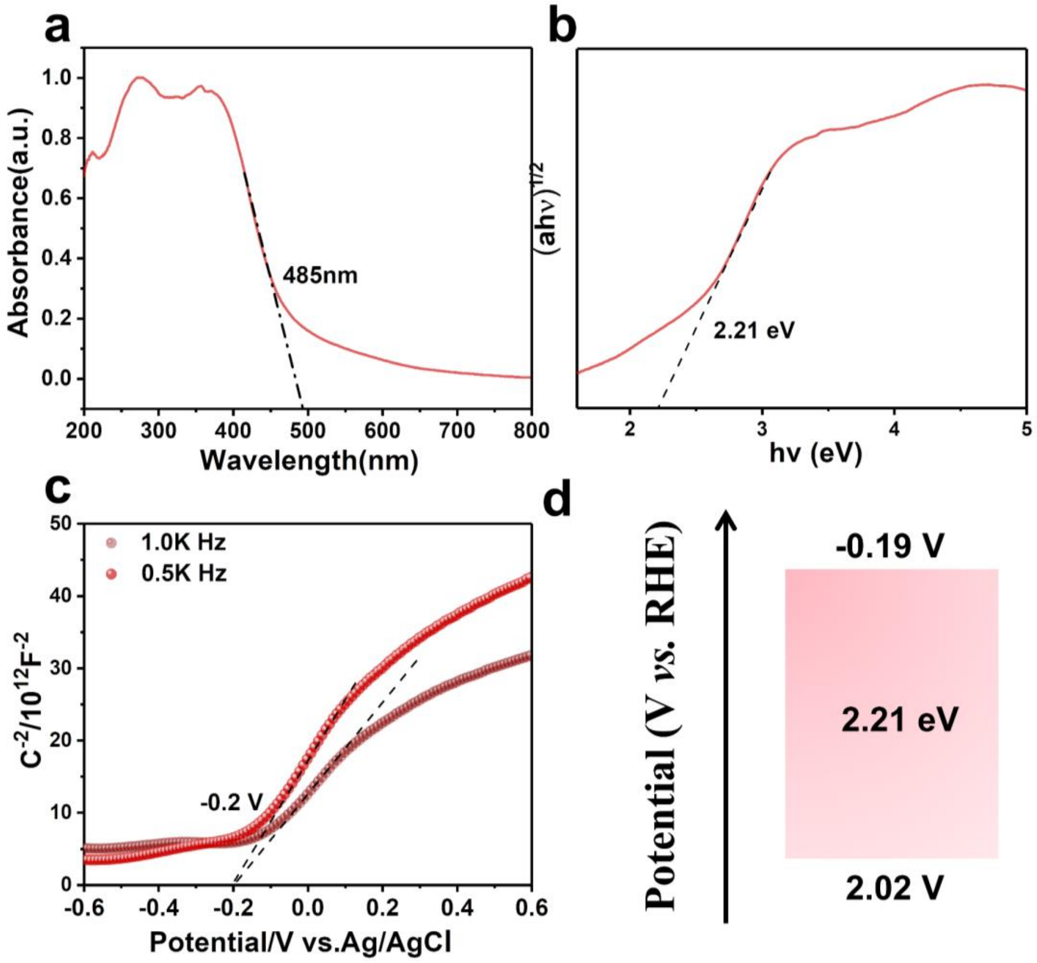

Figure S44 (a) UV-vis absorption spectrum and (b) corresponding Tauc plots of the BD-TPB; (c) Mott-Schottky plots of the BD-TPB in 0.5 M Na_2SO_4 solution with frequency of 0.5 kHz and 1.0 kHz; (d) Schematic illustration of the electronic band structures of the BD-TPB.

The oxidation-reduction potential of semiconductor photocatalyst can be described by energy band theory. The band of the semiconductor is discontinuous, a bandgap between the valence band (VB) and conduction band (CB). When it is irradiated by light whose energy is equal to or higher than the bandgap width of the photon, the electron in exciton on its VB will be excited to CB, and the corresponding hole will be produced on its VB. The potentials of the above holes and electrons determine the oxidation-reduction ability of the semiconductor under light excitation. Therefore, the oxidation-reduction ability of photocatalyst can be determined by the test of the semiconductor bandgap, VB, and CB potential.

UV-vis absorption spectrum was used to measure the light capture ability of PI-BD-TPB. As shown in **Figure S44a**, the absorption edge of PI-BD-TPB is ~ 485 nm. Tauc plots was calculated by the formula: $\alpha h\upsilon = A\ (h\upsilon - Eg)^{n/2}$, where α, h, ν, E_g and A are the absorption coefficient, Planck's constant, light frequency, band gap energy and a constant, respectively.

The n values of BD-TPB are 4, which is determined by the optical transition properties of a semiconductor (n = 1 for direct transition and n = 4 for indirect transition). The Tauc plot (**Figure S44b**) shows that the bandgap of the BD-TPB photocatalyst is 2.21 eV.

The Mott-Schottky plots of BD-TPB exhibited positive slopes, suggestive of their n-type semiconductor features (**Figure S44c**). A flat band potential of -0.20 V (*vs.* Ag/AgCl) was obtained for BD-TPB, namely +0.01 V (*vs.* RHE), respectively. A typical 3-electrodeconfiguration was used for the measurements. E_{CB} (V *vs.* RHE) and E_{VB} (V *vs.* RHE) are calculated according to the formula E_{CB} (V *vs.* RHE) = E_{fb} (V *vs.* Ag / AgCl) + 0.21 - X, and $E_{VB} = E_{CB} + E_g$), where X is from 0.1-0.2 V (the conduction bands of n-type semiconductors are normally 0.1-0.2 eV deeper than the flat-band potential) [26]. For BD-TPB photocatalyst, we used 0.2 V. Thus, E_{CB} is calculated to be -0.19 V (*vs.* RHE) and E_{VB} is 2.02 V (*vs.* RHE).

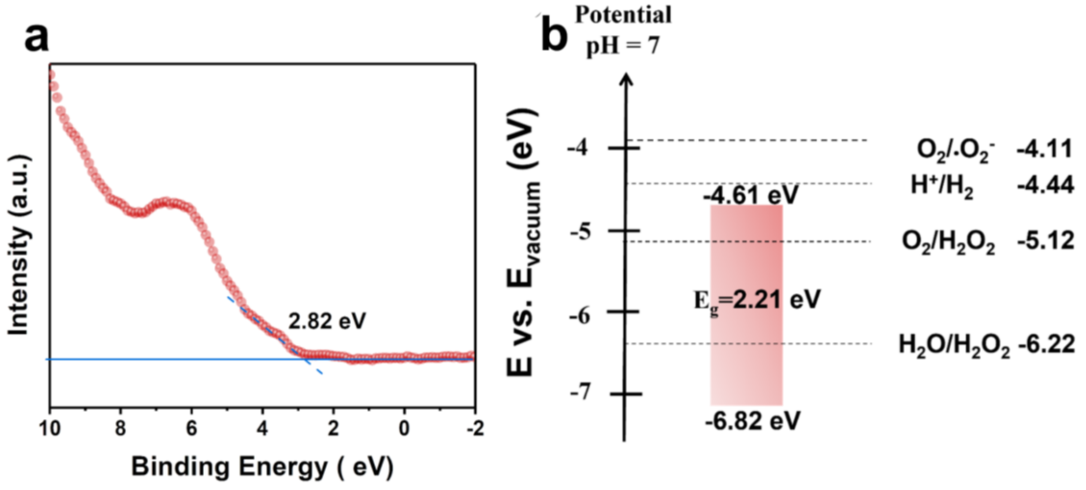

Figure S45 (a) X-ray photoelectron spectroscopy valence band spectra of the BD-TPB; (b) Calculated electronic band structures (relative to the vacuum level) of the BD-TPB.

Meanwhile, XPS valence band spectra were used to measure the valence band (VB) positions of BD-TPB, which revealed that the VB positions were -6.82 eV (relative to the vacuum energy level). -4.44 E vs. vacuum (eV) = 0 (V *vs.* RHE) [27]. The work functions (Φ) of X-ray photoelectron spectrometer were 4.0 eV. Therefore, combining the optical bandgaps and measured VB position, the band structure can be determined and illustrated in **Figure S45b**. Since the redox potentials for is -4.61 eV versus the vacuum level, namely 0.17 V (*vs.* RHE). Therefore, XPS valence band spectra were mutually verified with the Mott-Schottky method.

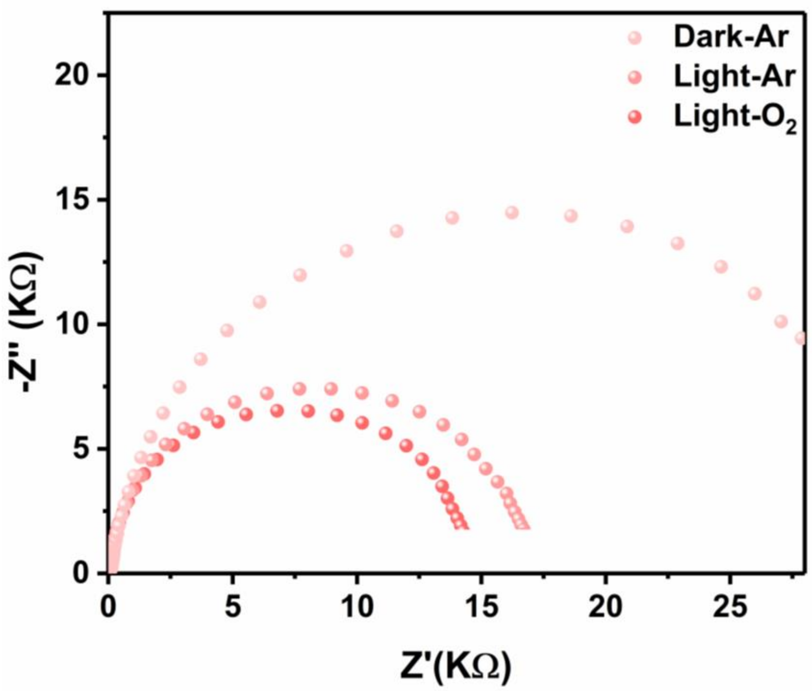

Figure S46 Electrochemical impedance spectroscopic (EIS) spectra of the BD-TPB.

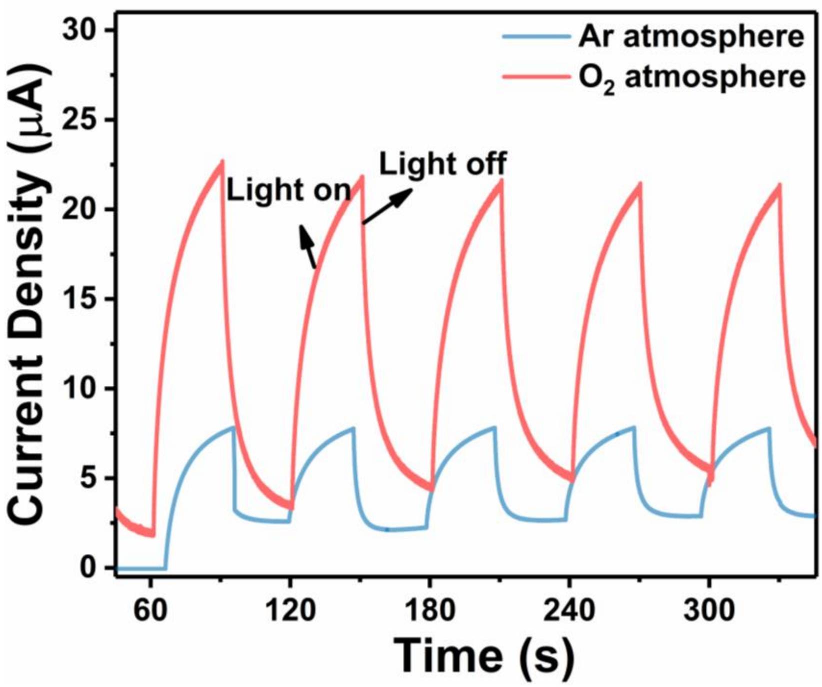

Figure S47 Photocurrent spectra of the PI-BD-TPB in Ar or O_2 atmosphere.

The EIS spectra showed the semicircle radius for BD-TPB in light or dark condition. The charge transfer resistance ratio under light irradiation for BD-TPB in O_2 atmosphere was smaller than that in Ar atmosphere, indicating the latter had a low charge transfer. The photocurrent of PI-BD-TPB in oxygen atmosphere was approximately four times higher than that in Ar atmosphere, indicating that the charge separation of photogenerated carriers was more efficient in O_2 atmosphere.

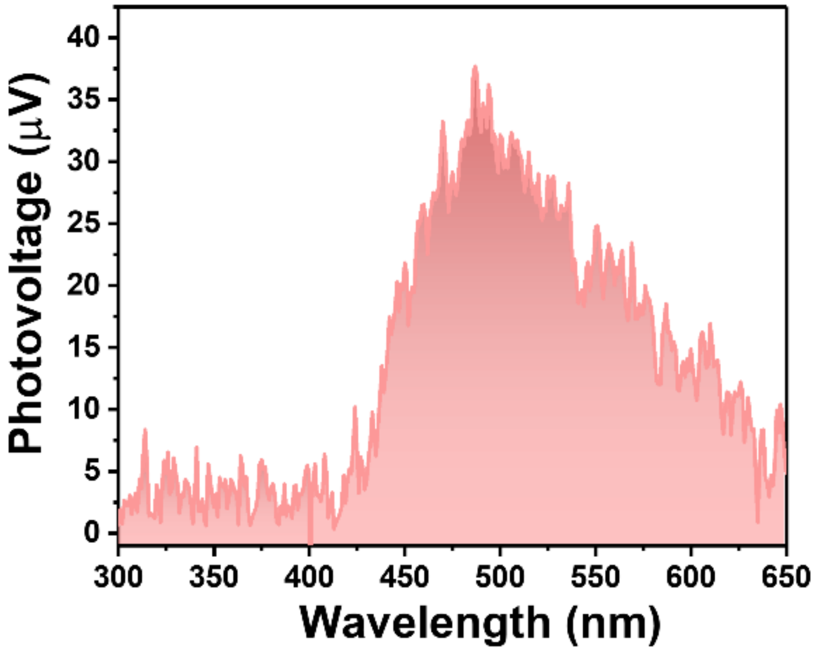

Figure S48 Surface photovoltage spectra of the PI-BD-TPB photocatalyst.

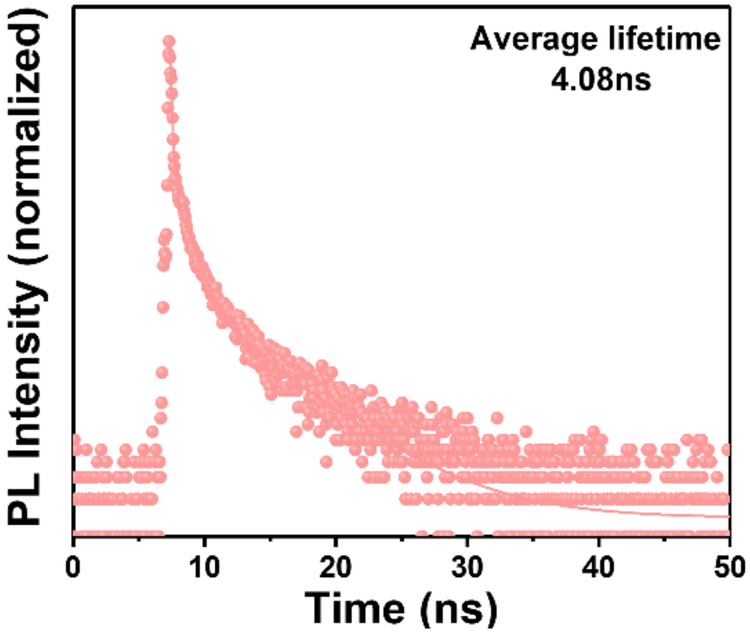

Figure S49 Transient fluorescence spectra of the PI-BD-TPB photocatalyst.

The photovoltage intensity of PI-BD-TPB was about 35.2 μV, showing that PI-BD-TPB can effectively enhanced charge separation. What's more, the average relaxation lifetime of PI-BD-TPB was 4.08 ns, displaying that PI-BD-TPB had prolonged lifetime of charge carrier. Combined with a series of photophysical characterizations, we confirm that PI-BD-TPB aerogel with donor-acceptor structure has the excellent charge separation efficiency and extended carrier lifetime.

4.3 EPR measurement for superoxide radical

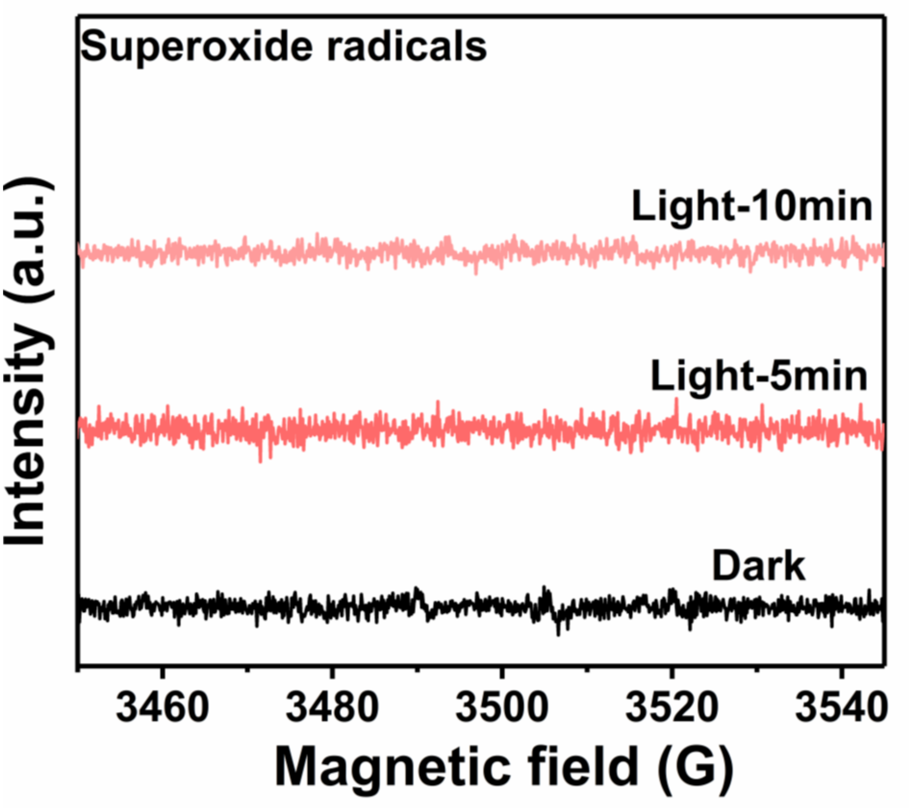

Figure S50 EPR signal for capturing superoxide radicals of the BD-TPB after 5 min and 10 min irradiation.

Superoxide radicals are captured by the mixture of 5,5-dimethyl-1-pyrroline N-oxide (DMPO) and CH_3OH. The use of DMPO and CH_3OH aims for capturing superoxide radicals as reported in the literature. In dark, no EPR signals were observed; EPR signals were also no observed after light irradiation. The result showed that O_2 was not directly reduced by photogenerated electron to form superoxide radical during the photocatalytic process.

4.4 Fluorescence measurement

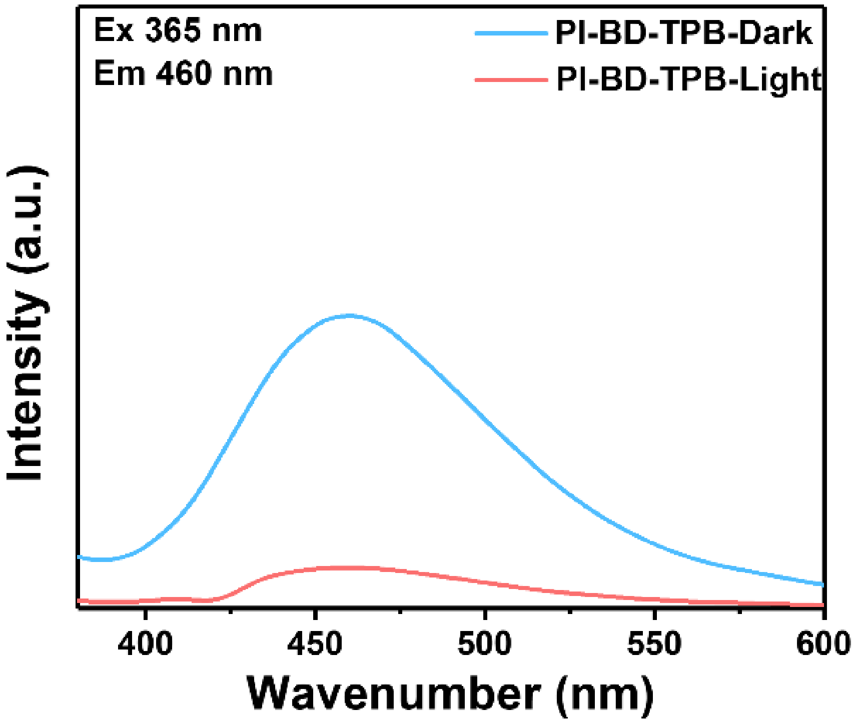

Figure S51 Fluorescence spectrum of BD-TPB and BD-TPB* anion radical in DMF solution with $Na_2S_2O_4$ as electron donor under Ar atmosphere in dark or light condition. (Excitation wavelength at 365 nm; emission wavelength at 460 nm).

In order to provide more evidence for the formation of the anion radical, fluorescence spectrum of BD-TPB and BD-TPB* anion radical in DMF were carried out. Compared to the BD-TPB, the fluorescence intensity of BD-TPB* radical anion was markedly decreased. Following process: $Na_2S_2O_4$ buffer solution (15 mM) was prepared freshly and then bubbled of Ar atmosphere for 30 min. Then, we injected it into the BD-TPB (1 mg, 15 mL) solution after a constant bubbling of Ar gas for 60 min. Fluorescence tests were performed in dark and again after light illumination.

Section 5. The mechanism of water oxidation

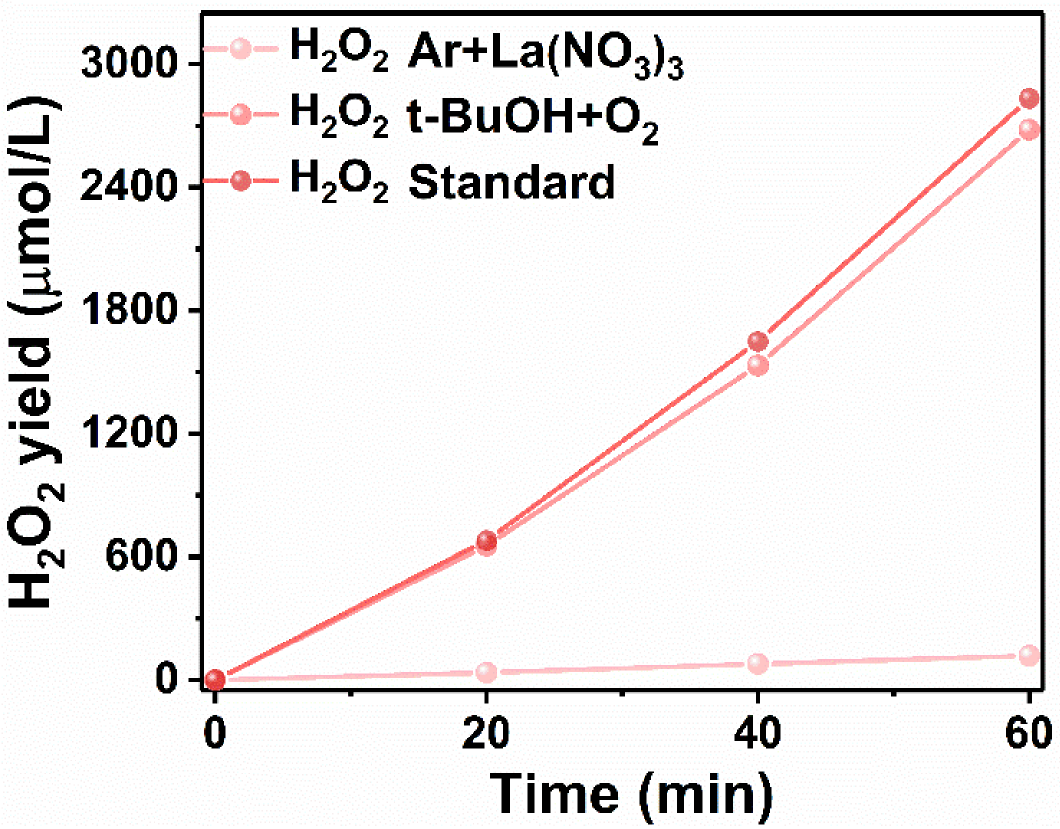

Figure S52 The photocatalytic H_2O_2 generation rate of the PI-BD-TPB under different reaction gases or different sacrificial agents.

The water oxidation half-reaction of BD-TPB was discussed in detail. We checked the role of the hole by using 2 mM La $(NO_3)_3$ as an electron scavenger. Since H_2O_2 was detected under Ar atmosphere for BD-TPB, confirming that the hole oxidized water to produce H_2O_2. In the presence of t-BuOH served as hydroxyl radical scavenger, the concentration of H_2O_2 production was not decreased significantly, indicating that hydroxyl radical was not key active specie in a two-electron water oxidation pathway.

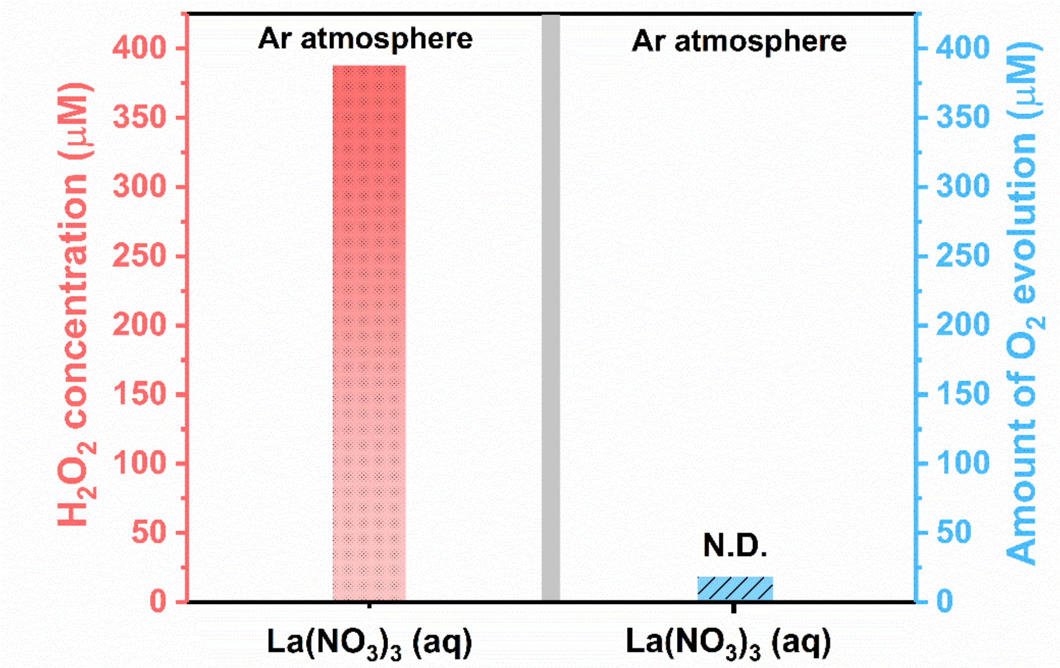

Figure S53 The photocatalytic H_2O_2 generation or O_2 production of the PI-BD-TPB in Ar-saturated water with 2 mM La $(NO_3)_3$. (Photocatalysts: 30 mg, Solution volume: 20 mL, Temperature: 298 K, Light source: 300 W Xe lamp with as the simulated sunlight.)

We checked the selectivity of water oxidation process by adding La $(NO_3)_3$ as electron scavenger to inhibit reduction reaction. H_2O_2 generation experiments were performed in Ar-saturated water with 2 mM La $(NO_3)_3$; O_2 evolution experiments were performed in Ar-saturated water with 2 mM La $(NO_3)_3$ and 50 mg La_2O_3 as pH buffer. Both experiments were performed in a sealed reactor for 4 h irradiation. According to these results, negligible O_2 was detected, while 0.388 mM H_2O_2 produced. It proved that photogenerated holes tended to oxidize water to produce H_2O_2 rather than O_2 in our system.

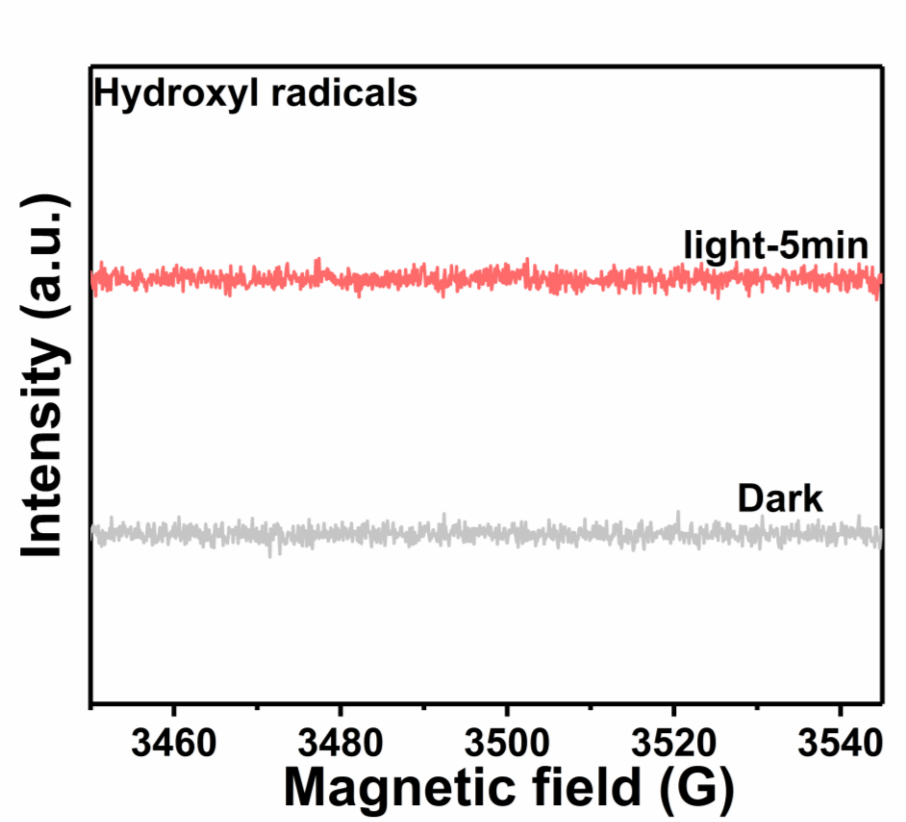

Figure S54 EPR signal for capturing hydroxyl radicals of the BD-TPB after irradiation.

Hydroxyl radical is captured by the mixture of DMPO and H_2O. In dark, no EPR signals were observed, and hydroxide radical was also not observed for BD-TPB after irradiation 5 min, showing the formation of hydroxyl radical and then H_2O_2 can be ruled out. We confirmed that H_2O_2 on BD-TPB was generated via a directly two-electron water oxidation pathway.

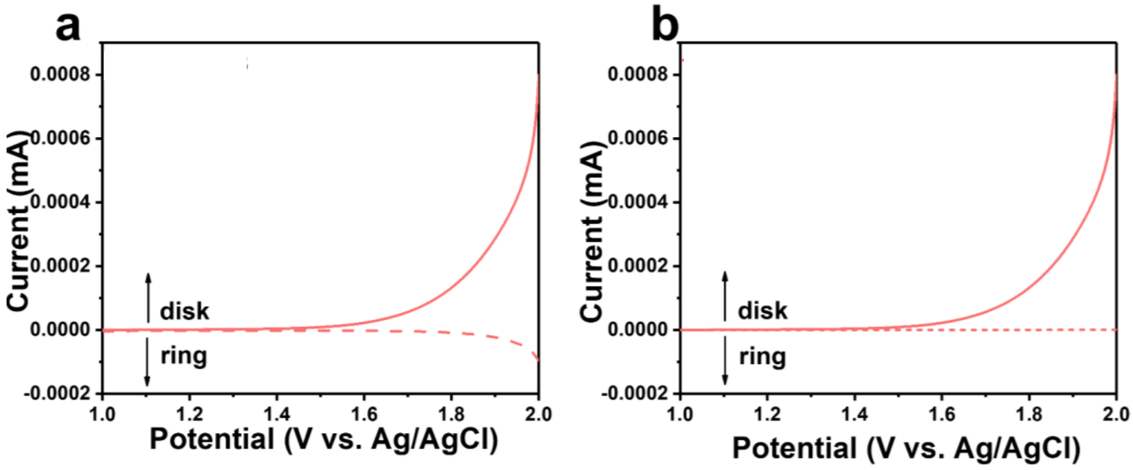

Figure S55 (a) and (b) Rotating ring disk electrode voltammograms obtained in 0.1 M phosphate buffer solution with a scan rate of 10 mV s^{-1} and a rotation rate of 1000 rpm. The potential of the Pt ring electrode is set at 0.6 V *vs* Ag/AgCl to detect H_2O_2 and set to -0.23 V *vs* Ag/AgCl to detect O_2.

The rotating ring disk electrode (RRDE) measurement was further explored the water oxidation reaction of PI-BD-TPB [8]. We setted the potential of the Pt ring electrode at -0.23 V or 0.6 V to detect O_2 or H_2O_2 production via water oxidation. In **Figure S55a**, when the potential was +0.6 V at the Pt ring electrode, an oxidation current was observed, showing that BD-TPB generated H_2O_2 via water oxidation. However, reduction current was not observed at the Pt ring electrode with a constant potential of -0.23 V, suggesting that PI-BD-TPB cannot generate O_2 via water oxidation (**Figure S55b**).

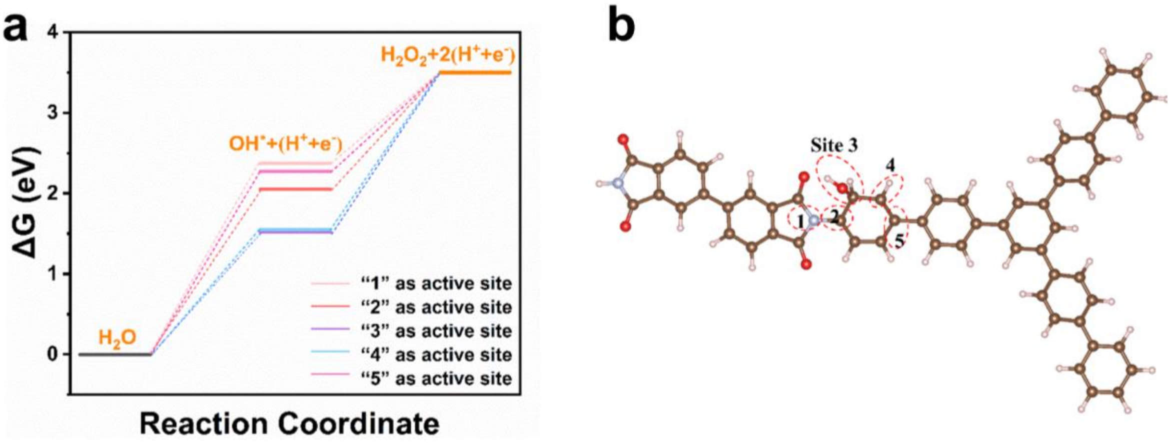

Figure S56 (a) Calculated free energy diagrams of two-electron water oxidization pathway toward H_2O_2 production on different active sites; (b) The adsorption energy of OH* on different sites of the BD-TPB.

For the two-electron water oxidation pathway, the largest Gibbs free energy step is the first oxidation step to form OH*[20]. Therefore, the formation of OH* in water oxidation process is crucial for the formation of H_2O_2. and the adsorption energy of the Site 3 (1.52 eV) was lowest (**Figure S56b**). The water oxidation most likely occurs at C atom in TPB unit due to the calculated OH* adsorption being negative for all sites.

Section 6. The mechanism of photocatalytic redox cycle

6.1 Isotopic labeling experiment

Table S2 Isotopic experiments with $^{18}O_2$ or $H_2{}^{18}O$ for H_2O_2 production after reaction.

Entry	O_2 source	H_2O source	Dark or Light	nH_2O_2 mmol	$nH_2{}^{18}O_2$ mmol	$n^{18}O/n^{16}O$ ppm
#1	$^{18}O_2$	$H_2{}^{16}O$	Dark	0	0	1988.1279
#2	$^{18}O_2$	$H_2{}^{16}O$	Light	0.1269	0.0652	2184.0358
#3	$^{16}O_2$	$H_2{}^{18}O$	Dark	0	0	1992.7183
#4	$^{16}O_2$	$H_2{}^{18}O$	Light	0.1040	0.0512	2146.3579

#1 as a blank control: the system of $H_2{}^{16}O$ and $^{18}O_2$, containing PI-BD-TPB polyimide aerogel photocatalyst; light source: dark; (**#1** and **#2** are controls for each other)

#2 as a control: the system of $H_2{}^{16}O$ and $^{18}O_2$, containing PI-BD-TPB polyimide aerogel photocatalyst; light source: 300 W Xe lamp with as the simulated sunlight;

#3 as a blank control: the system of $H_2{}^{18}O$ and $^{16}O_2$, containing PI-BD-TPB polyimide aerogel photocatalyst; light source: dark; (**#3** and **#4** are controls for each other)

#4 as a control: the system of $H_2{}^{18}O$ and $^{16}O_2$, containing PI-BD-TPB polyimide aerogel photocatalyst; light source: 300 W Xe lamp with as the simulated sunlight;

Experiment description: The reaction was carried out in a sealed reactor with first evacuated and then refilled with $^{18}O_2$ (97% atom ^{18}O purity, purchased from Wuxi Xinxiyi Technology Co., Ltd).) or $H_2{}^{18}O$ (97% atom ^{18}O purity, purchased from Beijing InnoChem Science & Technology Co., Ltd). We used powder photocatalysts and simulated sunlight as light sources. The reaction liquid after light irradiation was filtered with an injector topped with a 0.22 μm filter to obtain the hydrogen peroxide solution. ^{18}O in the produced $H_2{}^{18}O_2$ was determined by converting $H_2{}^{18}O_2$ to $H_2{}^{18}O$. First, appropriate amount of potassium titanium oxalate was added into the above solution to form the peroxy complex. The yellow powder was obtained by freeze-drying. Then the potassium titanium oxalate-H_2O_2 complex is re-dissolved by adding H_2O, and dried again to remove any remaining H_2O (for **#1** and **#2**) or $H_2{}^{18}O$ (for **#3** and **#4**). In particular, for water oxidation experiments, this process was repeated five times to ensure that $H_2{}^{18}O$ is removed, otherwise it interferes the results of the experiment (for **#3** and **#4**). Next, the KI solution (12 mL) reduced the potassium titanium oxalate-H_2O_2 complex to convert the contained H_2O_2 to H_2O. Finally, the converted H_2O was collected by distillation and detected by Liquid Water Isotope Analyzer. The ratio of $n^{18}O$ /$n^{16}O$ obtained directly from the Liquid Water Isotope Analyzer.

The amount of ^{18}O from water in **#1** and **#3** is calculated as follows:

Eq. 1……………………..$m(H_2O) = n^{18}O(H_2O) / n^{16}O(H_2O)$

Eq. 2……………………$nO(H_2O) = n^{16}O(H_2O) + n^{18}O(H_2O)$

where $n^{18}O(H_2O)$ is the ratio of ^{18}O in the H_2O; $n^{16}O(H_2O)$ is the ratio of ^{16}O in the H_2O; $m(H_2O)$ is the ratio between $n^{18}O(H_2O)$ and $n^{16}O(H_2O)$ (directly obtained from the measurements); and $nO(H_2O)$ is the total amount of O in the H_2O. $nO(H_2O)$ is 665.9 mmol in 12 mL KI solution. The average abundances of the oxygen isotopes ^{16}O and ^{18}O in nature are 99.762% and 0.200%, respectively.

By combining **Eq. 1** and **Eq. 2**, $n^{18}O(H_2O)$ and $n^{16}O(H_2O)$ can be calculated as:

Eq. 3……………………… $n^{18}O\ (H_2O) = (m(H_2O) \times nO\ )/\ (\ 1+ m(H_2O)\ )$

Eq. 4………………………$n^{16}O\ (H_2O) = nO\ /\ (1 + m(H_2O)\ )$

Accordingly, $n^{18}O(H_2O)$ and $n^{16}O(H_2O)$ is calculated as 1.321 mmol and 664.7 mmol, respectively, in **#1**, where $m(H_2O)$ is 1988.1279 ppm. And $n^{18}O(H_2O)$ and $n^{16}O(H_2O)$ is calculated as 1.324 mmol and 664.7 mmol, respectively. In **#3**, where $m(H_2O)$ is 1992.7183 ppm. The amount of ^{18}O in H_2O_2 in **#2** and **#4** is calculated based on the results in **#1** and **#3**, concurrently.

Eq. 5…..… $m= [n^{18}O\ (H_2O_2) + n^{18}O\ (H_2O)] / [n^{16}O\ (H_2O_2) + n^{16}O\ (H_2O)]$

Eq. 6…….…$nO(H_2O_2) = n^{16}O\ (H_2O_2) + n^{18}O\ (H_2O_2)$

By combining **Eq. 5** and **Eq. 6**, $n^{18}O\ (H_2O_2)$ can be calculated as:

Eq. 7…..…. $n^{18}O(H_2O_2) = (m \times (n^{16}O(H_2O)+nO)-n^{18}O) / (1 + m)$

Therefore, for **#2** ($^{18}O_2$ + $H_2{}^{16}O$), the amount of O in H_2O_2 (0.2539 mmol, C_{H2O2}=6.3466 mmol/L, Vaq=20 mL) is calculated based on the concentration of H_2O_2, and m is measured to be 2184.0358 ppm. Then $n^{18}O\ (H_2O_2)$ is calculated to be 0.1304 mmol, and thus the amount of $H_2{}^{18}O_2$ is 0.0652 mmol. The total H_2O_2 production amount is measured to be 0.1269 mmol. Therefore, half of the H_2O_2 is mainly produced via the electron reduction reaction.

For **#4** ($^{16}O_2$ + $H_2{}^{18}O$), the amount of O in H_2O_2 (0.2080 mmol, C_{H2O2}=5.2006 mmol/L, Vaq=20 mL) is calculated based on the concentration of H_2O_2, and m is measured to be 2146.3579 ppm. Then $n^{18}O(H_2O_2)$ is calculated to be 0.1023 mmol, and thus the amount of $H_2{}^{18}O_2$ is 0.0512 mmol. The total H_2O_2 production amount is measured to be 0.1040 mmol. Therefore, near half of H_2O_2 production is mainly produced via the hole oxidation reaction.

6.2 *In situ* FTIR analysis

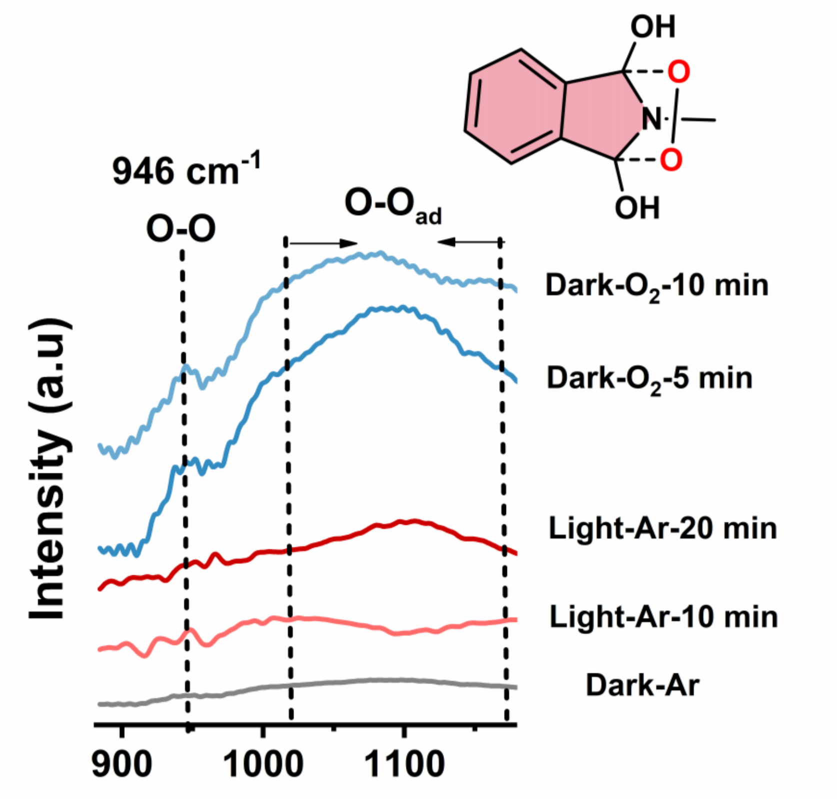

Figure S57 *In situ* FTIR spectrum of the BD-TPB with Ar atmosphere under 300 W Xe lamp and then O_2 gas purging in the dark at 900-1200 cm^{-1}.

6.3 Elemental analysis

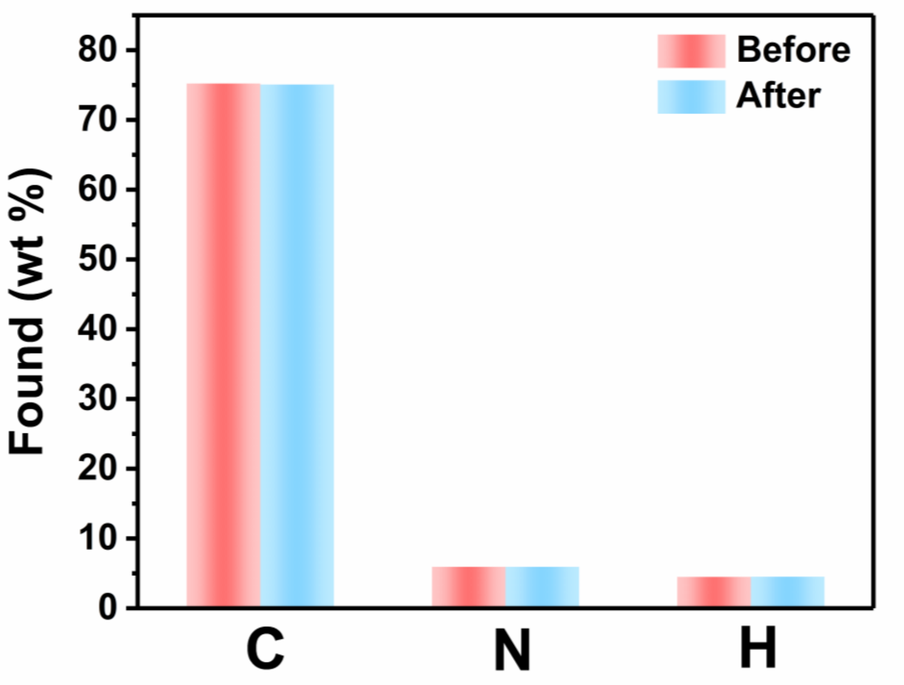

Figure S58 Elemental analysis of the BD-TPB photocatalyst after photoreaction [26].

6.5 H_2O_2 production on various PI photocatalysts with carbonyl group

#A photocatalyst with carbonyl groups (PAA photocatalyst)

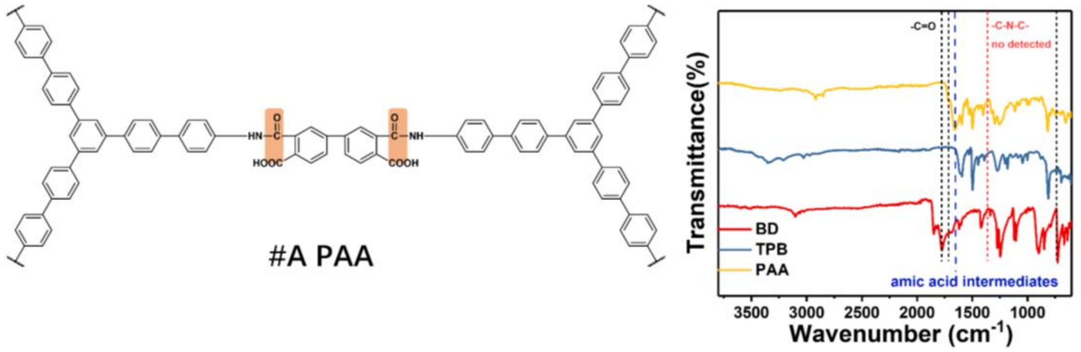

Figure S59 Structure diagram (right) and FTIR spectra (left) of the PAA photocatalyst.

#B1-3 photocatalyst with carbonyl groups (LP photocatalyst)

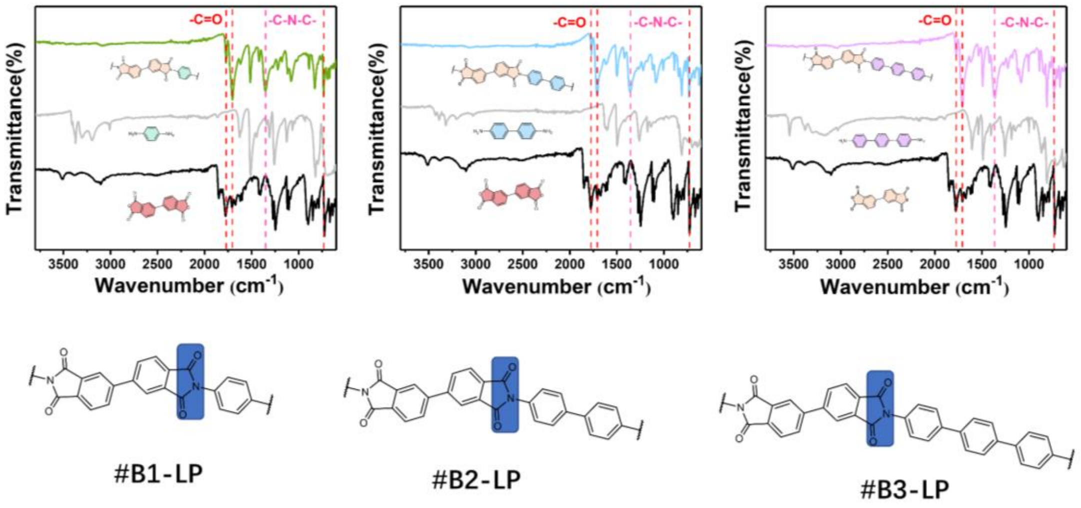

Figure S60 Structure diagram (up) and FTIR (on) spectra of linear polymer photocatalysts.

#C1-2 photocatalyst with carbonyl groups (CP photocatalyst)

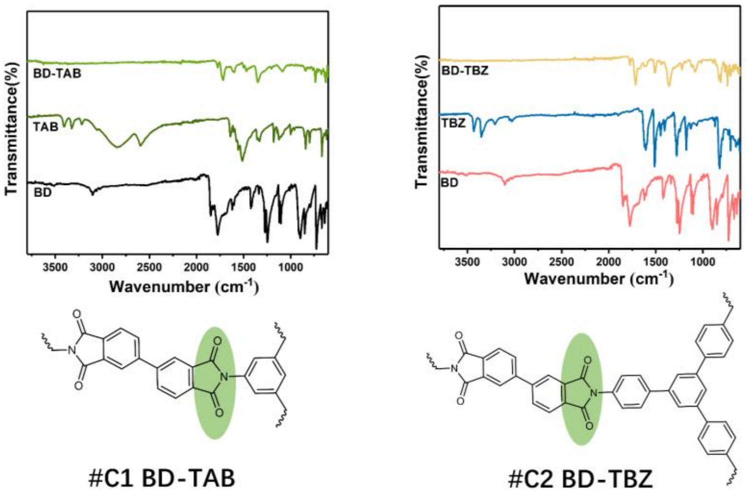

Figure S61 Structure diagram (up) and FTIR (on) spectra of cross-linked polymer.

#D photocatalyst with carbonyl groups (DA-TABPB photocatalyst)

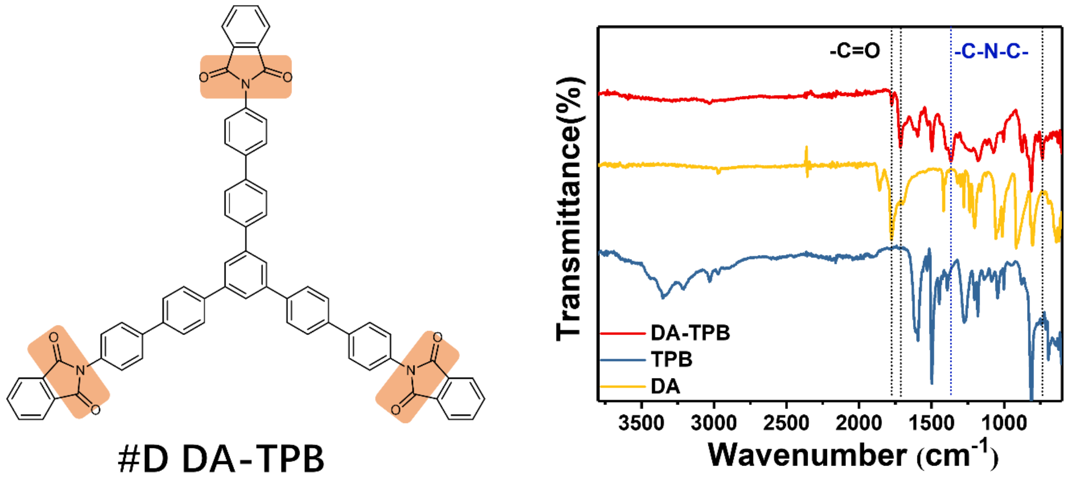

Figure S62 Structure diagram (right) and FTIR spectra (left) of the DA-TABPB photocatalyst.

#E photocatalyst with carbonyl groups (HCP photocatalyst)

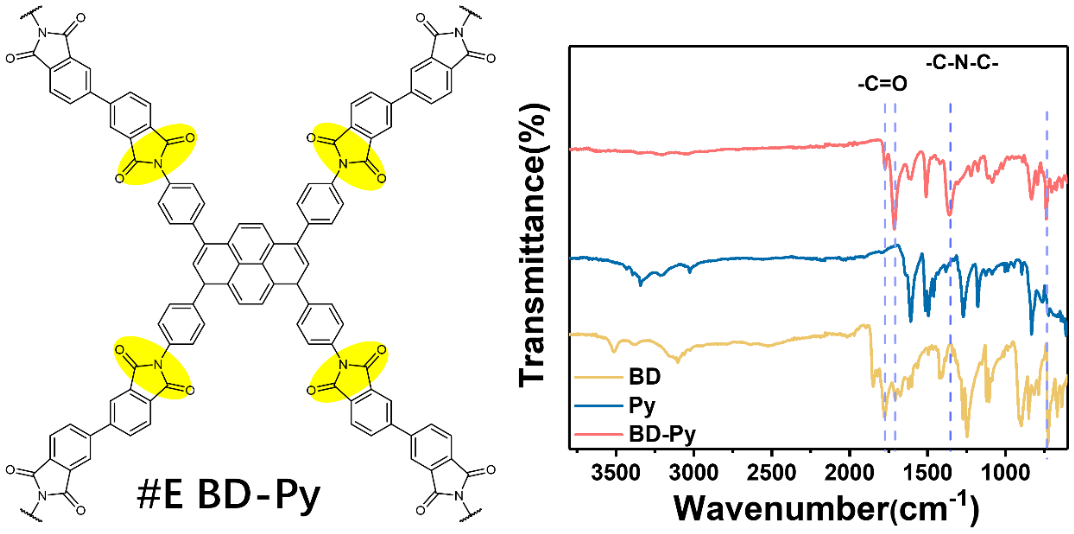

Figure S63 Structure diagram (right) and FTIR spectra (left) of hyper-crosslinked polymer photocatalyst with hyper-conjugated framework.

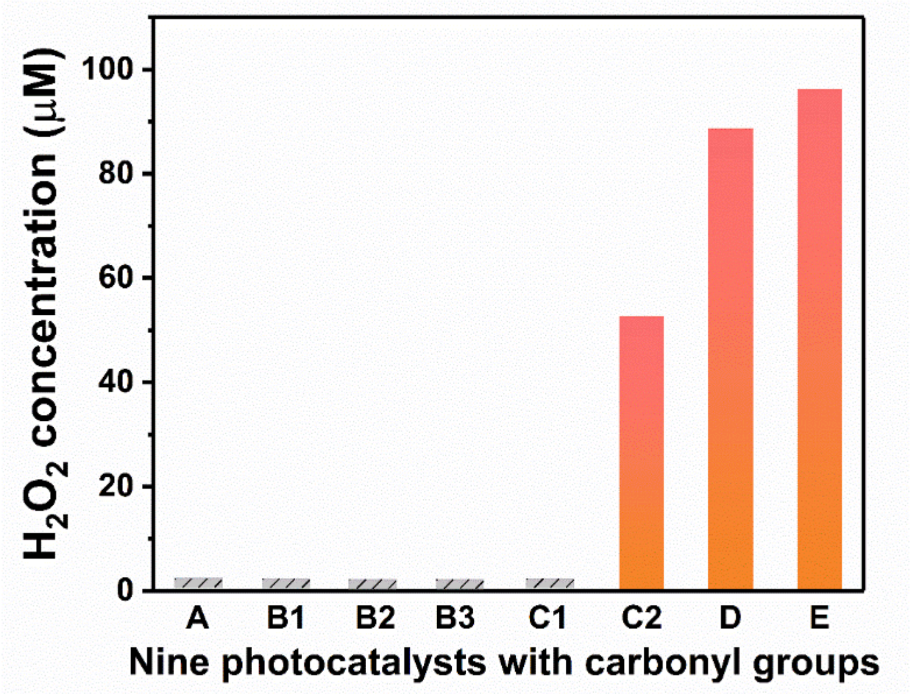

Figure S64 H_2O_2 production in 5 mM vitamin C solution as electron donor in argon atmosphere on nine photocatalysts with carbonyl group under "Dark-O_2" condition. Reaction conditions: 20 mg photocatalyst, 15 mL solution, 300 W Xe lamp.

The key experiment to verify the mechanism of anion radical intermediate-mediated H_2O_2 synthesis is that H_2O_2 can be produced in the dark reaction. Nine photocatalysts with carbonyl group were prepared and evaluated, respectively. We preliminarily confirmed the structure of nine photocatalysts by FTIR spectroscopy. H_2O_2 generation was produced on nine photocatalysts with carbonyl group in 5 mM vitamin C solution in argon atmosphere. Oxygen quickly was kept bubbling to the closed system for 30 min after light irradiation for 60 min.

No H_2O_2 was generated by PAA photocatalyst under "Dark-O_2" condition, showing that imide ring played a crucial part in anion radical intermediate-mediated H_2O_2 production. Similarly, no H_2O_2 production under "Dark-O_2" condition was detected by three linear polyimide (B1-LP, B2-LP and B3-LP). This result suggests that crosslinked framework structures also play an important role in the formation of anion radical intermediate. Interestingly, we detected H_2O_2 **#C2** photocatalyst after "Dark-O_2" reaction, but no H_2O_2 was produced in **#C1** photocatalyst. We further speculate that hyper-conjugated donor unit not only promotes charge separation, but also facilitates stabilization of anion radical intermediate. To test our hypothesis, we designed and synthesized **#D** photocatalyst (DA-TABPB) and **#E** photocatalyst (BD-Py) with hyper-conjugated donor units. Hydrogen peroxide was also detected after "Dark-O_2" reaction in both **#D1** and **#E1** photocatalyst.

6.4 Electrochemical measurement

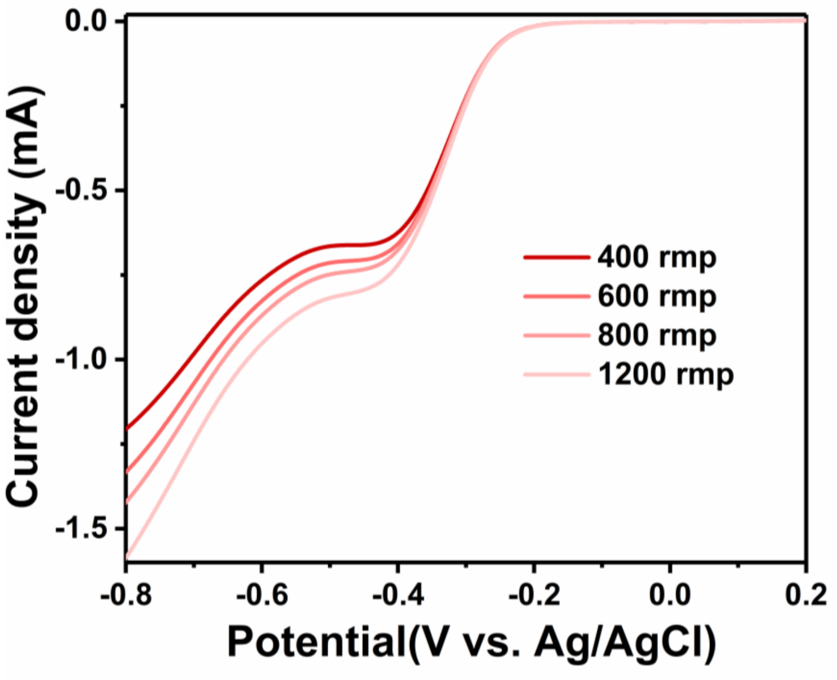

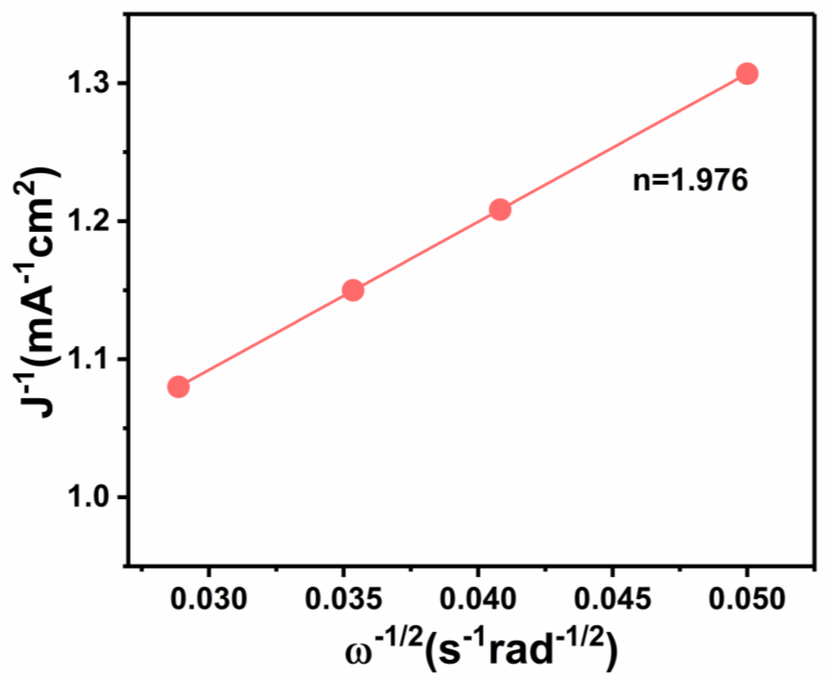

Figure S65 Linear-sweep rotating disk electrode (RDE) voltammograms of the PI-BD-TPB aerogel measured at different rotating speeds and the corresponding Koutecky-Levich plot [19].

The average electron transfer number was calculated to be 1.976 and the corresponding potential at -0.6 V (vs. Ag / AgCl).

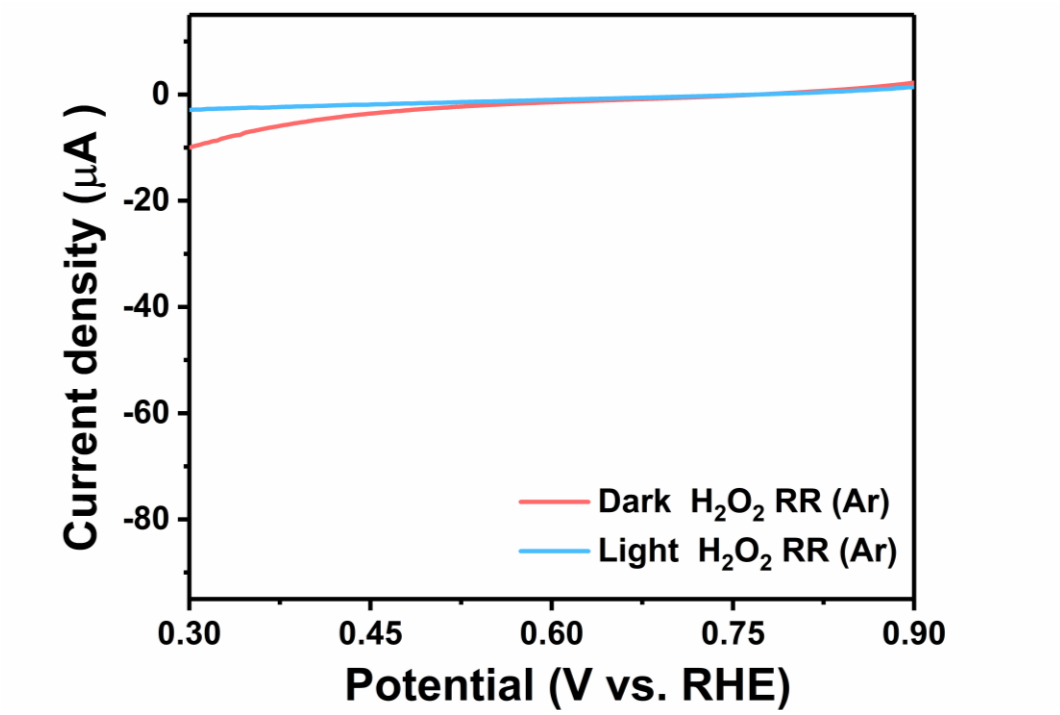

Figure S66 The linear sweep voltammetry curves of H_2O_2 reduction reaction recorded in Ar-saturated 0.5 M H_2SO_4 electrolyte containing 15 mM H_2O_2 at a scan rate of 5 mV s^{-1} under light irradiation.

To investigate the O_2 reduction reaction pathway, the electrochemical H_2O_2 reduction reaction (H_2O_2 RR) measurements were conducted in Ar-saturated 0.5 M H_2SO_4 electrolyte containing 15 mM H_2O_2.[28] The glassy carbon electrode, platinum wire and Ag/AgCl electrode were employed as the working electrode, the counter electrode and the reference electrode, respectively. As presented in **Fig. S66**, it demonstrated that BD-TPB under light continuous irradiation had a poor H_2O_2 reduction reaction activity due to a negligible current density.

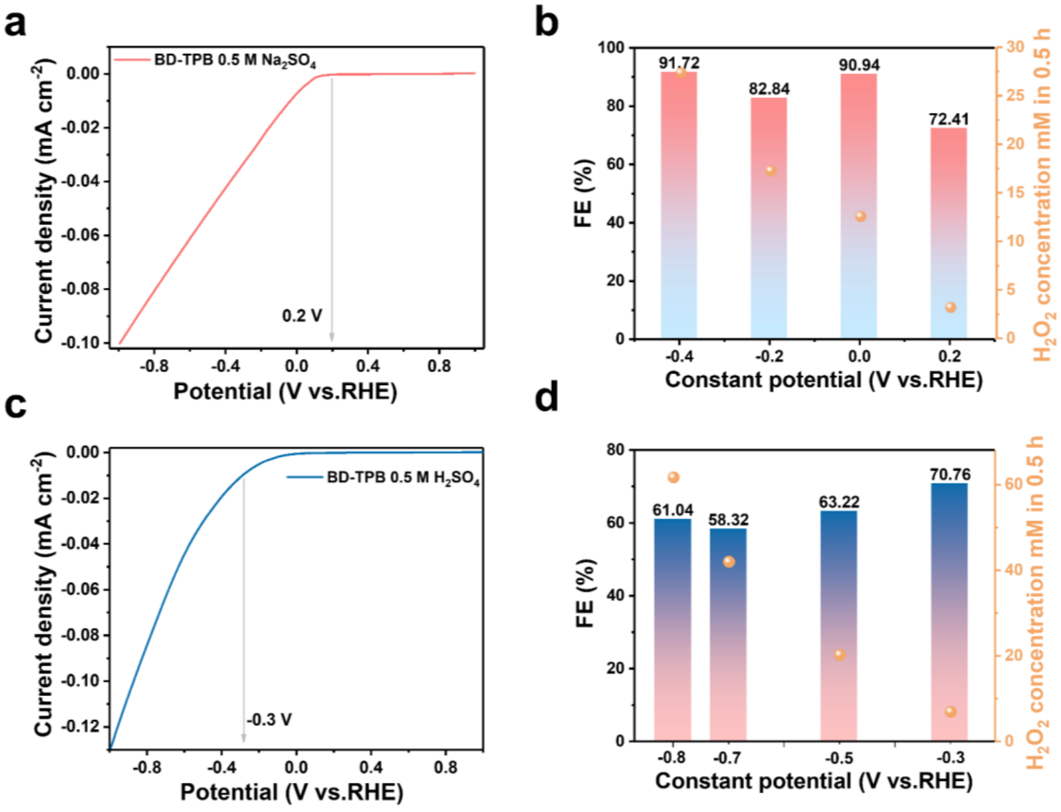

Figure S67. Electrocatalytic performance for the PI-BD-TPB catalyst toward two-electron oxygen reduction for H_2O_2 electrosynthesis in neutral and acidic electrolyte. (a) The linear sweep voltammetry curve of the PI-BD-TPB catalyst obtained under 50 mV s^{-1} in O_2-saturated 0.5 M Na_2SO_4; (b) H_2O_2 concentration and the corresponding faradic efficiency (FE) % of the PI-BD-TPB catalyst at different applied potentials in the flow cell device with O_2-saturated 0.5 M Na_2SO_4; (c) LSV curve of the PI-BD-TPB catalyst obtained under 50 mV s^{-1} in O_2-saturated 0.5 M H_2SO_4; (d) H_2O_2 concentration and the corresponding FE % of the PI-BD-TPB catalyst at different applied potentials in the flow cell device with O_2-saturated 0.5 M H_2SO_4.

We tested the electrochemical performance for H_2O_2 generation on the PI-BD-TPB photocatalyst via constant potential method using different electrolyte solutions. We measured the concentration of H_2O_2 and calculated the corresponding faradic efficiency (FE) in the flow cell device with O_2-saturated 0.5 M Na_2SO_4 solution as neutral electrolyte or 0.5 M H_2SO_4 solution as acidic electrolyte. We used the PI-BD-TPB/carbon paper (1.0 cm^{-2}) as working electrode, Ag/AgCl electrode as the reference electrode and platinum wire electrode as the counter electrode, respectively. 0.5 M Na_2SO_4 solution and 0.5 M H_2SO_4 solution were employed as neutral and acidic electrolyte for O_2 reduction reaction, respectively. Hydrogen peroxide was formed in the electrolyte solution. We took the electrolyte solution and determined the concentration of H_2O_2 by potassium titanium oxalate method. The FE for H_2O_2 production is calculated according to the following equation:

$$FE(\%) = \frac{N \times F \times n_{H_2O_2}}{Q} \times 100\% \ldots\ldots\ldots\ldots\ldots\ldots$$

where N is the number of charge transfer for H_2O_2 production ("2" in the reaction), F is the Faraday constant (96485 C mol^{-1}), n_{H2O2} is the produced H_2O_2 (mol), and Q (C) is the charge transfer number for the whole reaction, which can be obtained from the I-t curves.

The linear sweep voltammetry curves in **Figure S67a** and **S67c** exhibited that the currents of PI-BD-TPB catalyst recorded in the O_2-saturated with 0.5 M Na_2SO_4 solution or 0.5 M H_2SO_4 solution, respectively. Due to the significant oxygen reduction current at 0.2 V vs. RHE in 0.5 M Na_2SO_4 neutral electrolyte, constant potential tests were carried out at 0.2 V, 0.0 V, -0.2 V and -0.4 V vs. RHE applied potentials. In neutral electrolyte, the H_2O_2 concentration at the cathode side was measured to be 3.09 mM, 12.49 mM, 17.21 mM and 27.33 mM at the corresponding potentials of 0.2 V, 0.0 V, -0.2 V and -0.4 V vs. RHE, respectively. Therefore, the corresponding FE% were 72.41%, 90.94%, 82.84% and 91.72%, respectively (**Figure S67b**). Similarly, constant potential tests were carried out at -0.3 V, -0.5 V, -0.7 V and -0.8 V vs. RHE potentials in 0.5 M H_2SO_4 acidic electrolyte due to the significant oxygen reduction current at -0.3 V vs. RHE. The H_2O_2 concentration at the cathode side was measured to be 6.91 mM, 20.23 mM, 41.95 mM and 61.67 mM in acidic electrolyte at the corresponding potentials of -0.3 V, -0.5 V, -0.7 V and -0.8 V vs. RHE, respectively. The FE was calculated to 70.76%, 63.22%, 58.32% and 61.04% at the corresponding potential (**Figure S67d**). Therefore, the above results show that PI-BD-TPB photocatalyst has excellent two-electron oxygen reduction activity for H_2O_2 electrosynthesis, excluding the possibility of the surface charge of the catalyst varies with the electrode potential.

Section 7. Theoretical simulations and computation

Computational Methods

All the spin-polarized density functional theory (DFT) calculations were performed by using the Vienna ab initio simulation package (VASP) [29-30]. The D3 correction method (DFT-D3) was employed in order to include van der Waals (vdW) interactions [31]. The projector-augmented wave (PAW) method was used to represent core-valence interactions [32]. Valence electrons were described by a plane wave basis with an energy cutoff of 400 eV. The generalized gradient approximation with the Perdew-Burke-Ernzerhof (GGA-PBE) functional was used to model electronic exchange and correlation [33]. Electron smearing was employed via Gaussian smearing method with a smearing width consistent to 0.05 eV. The conjugate gradient algorithm was used in geometry optimization calculations. Optimized structures were obtained by minimizing the forces on each ion until they were less than 0.03 eV/Å. The energy convergence criteria were set to 10^{-5} eV.

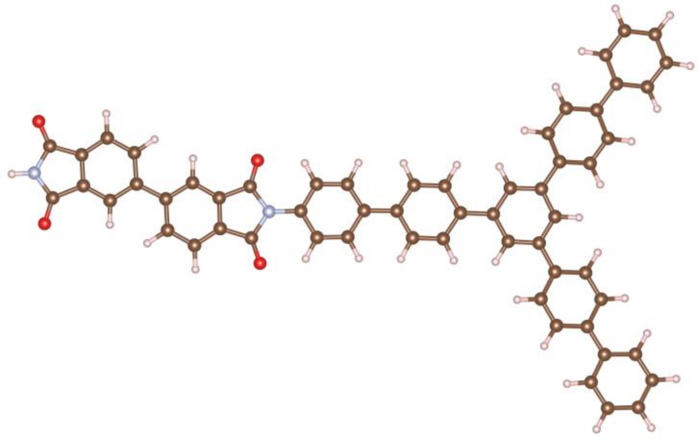

Figure S68 The structural model of BD-TPB, modeled by cutting the supercell obtained from the 2D optimized structure of the BD-TPB model.

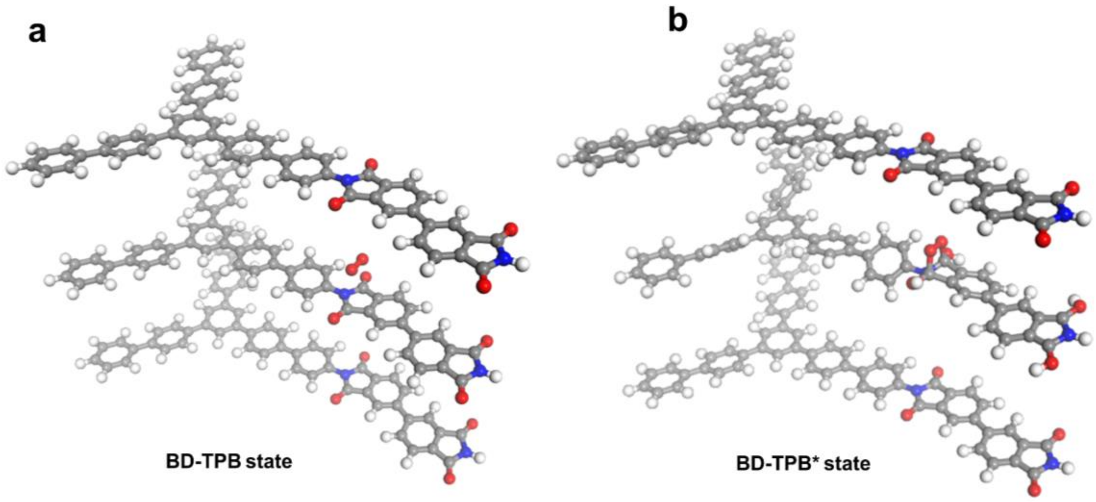

Figure S69 (a) The structural model of -C=O state (BD-TPB) and (b) radical anion state (BD-TPB*) of O_2 adsorption using a three-layer cluster model.

Geometry optimization of structural unit saturated with hydrogen atoms was performed by density functional theory (DFT) using CAM-B3LYP exchange-correlation functional [34] in conjunction with 6-31G(d) basis set [35]. Vibrational frequency analyse was carried out at the same level on the optimized structure to determine its nature. Electron vertical excitation calculation for studied molecule was conducted with the time-dependent DFT [36-37] method at the CAM-B3LYP/6-31G(d) level of theory. All quantum chemistry calculations were implemented by Gaussian 09 (A.03) program package [38]. The isosurface maps of hole and electron distributions, highest occupied molecular orbital (HOMO) and lowest unoccupied molecular orbital (LUMO), and molecular electrostatic potential (ESP) were all rendered by means of Visual Molecular Dynamics (VMD) software [39] based on the related files exported by Multiwfn 3.8(dev) code [40].

Table S3 Optimized Cartesian coordinates for studied molecule at the CAM-B3LYP/6-31G(d) level of theory, the values are given in Å

atom	*x*	*y*	*z*
C	13.88516725	0.34792560	-0.20915707
C	14.10149612	0.81420261	1.08077311
C	13.08353362	0.82982432	2.01394056
C	11.83538060	0.35995311	1.61250458
C	11.60320382	-0.11300091	0.31202153
C	12.65343470	-0.11739341	-0.61758412
C	15.16251545	0.46461344	-0.97556748
C	15.52611414	1.24470928	1.19652440
H	13.25542997	1.18882336	3.02289749
H	11.02194129	0.33880361	2.33034856
H	12.50320218	-0.45327075	-1.63807253
O	15.38907311	0.16785242	-2.12235875
O	16.10791733	1.70310556	2.14803988
N	16.07432230	1.00242897	-0.06597051
C	10.25633341	-0.60242627	-0.07493082
C	10.12571384	-1.72855119	-0.90247165
C	9.10456955	0.05515240	0.38103524
C	8.88080399	-2.21738454	-1.29064880
H	11.02245343	-2.24324048	-1.23166226
C	7.87827822	-0.44054982	-0.00813303
H	9.16753408	0.94205425	1.00237368
C	7.76324879	-1.55100894	-0.82717251
H	8.78751269	-3.09094443	-1.92662428
C	6.51036948	0.05155669	0.31482499
C	6.31857628	-1.81748778	-1.06171850
O	6.20554476	0.99350664	1.00416805
O	5.82262453	-2.69526729	-1.72372969

N	5.62235454	-0.81876776	-0.34811597
C	4.20374716	-0.70595219	-0.30300861
C	3.44861171	-0.93802548	-1.44981479
C	3.56857043	-0.36320435	0.88792278
C	2.06731499	-0.83228400	-1.39664122
H	3.93906741	-1.20581104	-2.37645189
C	2.18737502	-0.24885614	0.92243338
H	4.15263799	-0.18083976	1.78042782
C	1.40917628	-0.48333855	-0.21431961
H	1.70273231	0.00005580	1.86091615
H	1.49132191	-0.99652244	-2.30152175
C	-0.06744278	-0.36551830	-0.16741138
C	-0.88496503	-1.25416780	-0.87103837
C	-0.68756196	0.63808611	0.58185004
C	-2.26653999	-1.14349961	-0.82755594
H	-0.43249047	-2.05403884	-1.44893376
C	-2.06912866	0.74752617	0.62626116
H	-0.07834965	1.35371861	1.12520249
C	-2.88675374	-0.14077758	-0.07741895
H	-2.52314930	1.52793648	1.22901954
H	-2.87517382	-1.83830032	-1.39780245
C	-4.36486756	-0.02240763	-0.02778434
C	-5.16851679	-1.16187547	0.02436521
C	-4.97960967	1.23028807	-0.04886547
C	-6.56031545	-1.06760804	0.05696131
H	-4.70183020	-2.14142906	0.04022605
C	-6.36906338	1.35731700	-0.01684775
H	-4.36684229	2.12125120	-0.13960993
C	-7.14578747	0.19906473	0.03682472

H	-8.22481063	0.28633160	0.11166545
C	-7.00731227	2.69624191	-0.03929802
C	-6.45260243	3.77240436	0.65856255
C	-8.18331451	2.92342691	-0.75920454
C	-7.04912171	5.02402109	0.63735814
H	-5.55705973	3.61884527	1.25265367
C	-8.77994228	4.17499341	-0.78037704
H	-8.62014467	2.11486440	-1.33707360
C	-8.22526371	5.25141593	-0.08256113
H	-6.61232273	5.83245072	1.21539373
H	-9.67520805	4.32864766	-1.37482849
C	-7.39999084	-2.28857707	0.12910938
C	-8.60021395	-2.38047564	-0.58069227
C	-7.01738621	-3.38432969	0.90744790
C	-9.38546779	-3.52153785	-0.51551339
H	-8.90778012	-1.55727972	-1.21816528
C	-7.80297942	-4.52522625	0.97276185
H	-6.10672895	-3.32992776	1.49586888
C	-9.00315956	-4.61776434	0.26260549
H	-10.29635482	-3.57597358	-1.10357575
H	-7.49588839	-5.34753147	1.61149735
C	-8.86323478	6.59004410	-0.10533974
C	-10.25467635	6.72190904	-0.06970987
C	-8.08864375	7.75256507	-0.16303413
C	-10.85280571	7.97514904	-0.09176219
H	-10.87266716	5.83203166	-0.00008060
C	-8.68534010	9.00650973	-0.18372842
H	-7.00741027	7.67038295	-0.21609311
C	-10.07038773	9.12309758	-0.14852886

H	-11.93508071	8.05498903	-0.05599858
H	-8.06509224	9.89618400	-0.23603840
C	-9.84294877	-5.83817430	0.33243882
C	-11.23775073	-5.74785988	0.36650927
C	-9.26018016	-7.10862680	0.36591051
C	-12.02480663	-6.89032213	0.43114299
H	-11.70856152	-4.76960889	0.36620150
C	-10.04591529	-8.25191525	0.43199859
H	-8.17960275	-7.20070755	0.31586155
C	-11.43200148	-8.14756144	0.46451171
H	-13.10609874	-6.79704771	0.46403795
H	-9.57278849	-9.22905490	0.44983325
H	17.03838662	1.19702064	-0.29720137
H	-10.53734627	10.10294996	-0.16524642
H	-12.04667155	-9.04087397	0.51564385

Section 8. Supplementary References

1. Pan, C. *et al.* Efficient and stable H_2O_2 production from H_2O and O_2 on $BiPO_4$ photocatalyst. *Appl. Catal. B Environ.* **316**, 121675 (2022).
2. Wang, X. *et al.* Ambient preparation of benzoxazine-based phenolic resins enables long-term sustainable photosynthesis of hydrogen peroxide. *Angew. Chem. Int. Ed.* **62**, e202302829 (2023).
3. Liu, Y. *et al.* Substoichiometric covalent organic frameworks with uncondensed aldehyde for highly efficient hydrogen peroxide photosynthesis in pure water. *Appl. Catal. B Environ.* **331**, 122691 (2023).
4. Yue, J.-Y. *et al.* Thiophene-containing covalent organic frameworks for overall photocatalytic H_2O_2 synthesis in water and seawater. *Angew. Chem. Int. Ed.* **62**, e202309624 (2023).
5. Liu, F. *et al.* Covalent organic frameworks for direct photosynthesis of hydrogen peroxide from water, air and sunlight. *Nat. Commun.* **14**, 4344 (2023).
6. Mou, Y. *et al.* Linkage microenvironment of azoles-related covalent organic frameworks precisely regulates photocatalytic generation of hydrogen peroxide. *Angew. Chem. Int. Ed.* **62**, e202309480 (2023).
7. Ma, J. *et al.* Extended conjugation tuning carbon nitride for non-sacrificial H_2O_2 photosynthesis and hypoxic tumor therapy. *Angew. Chem. Int. Ed.* **61**, e202210856 (2022).
8. Kou, M. *et al.* Molecularly engineered covalent organic frameworks for hydrogen peroxide photosynthesis. *Angew. Chem. Int. Ed.* **61**, e202200413 (2022).
9. Wu, C. *et al.* Polarization engineering of covalent triazine frameworks for highly efficient photosynthesis of hydrogen peroxide from molecular oxygen and water. *Adv. Mater.* **34**, 2110266 (2022).
10. Chai, S. *et al.* Rational design of covalent organic frameworks for efficient photocatalytic hydrogen peroxide production. *Environ. Sci.: Nano.* **9**, 2464-2469 (2022).
11. Xu, L., Liu, Y., Li, L., Hu, Z. & Yu, J. C. Fabrication of a photocatalyst with biomass waste for H_2O_2 synthesis. *ACS Catal.* **11**, 14480-14488 (2021).
12. Ye, Y.-X. *et al.* A solar-to-chemical conversion efficiency up to 0.26% achieved in ambient conditions. *Proc. Natl. Acad. Sci. U. S. A* **118**, e2115666118 (2021).
13. Li, L., Xu, L., Hu, Z. & Yu, J. C. Enhanced mass transfer of oxygen through a gas–liquid–solid interface for photocatalytic hydrogen peroxide production. *Adv. Funct. Mater.* **31**, 2106120 (2021).
14. Liu, L. *et al.* Linear conjugated polymers for solar-driven hydrogen peroxide production: the importance of catalyst stability. *J. Am. Chem. Soc.* **143**, 19287-19293 (2021).
15. Cheng, H. *et al.* Rational design of covalent heptazine frameworks with spatially separated redox centers for high-efficiency photocatalytic hydrogen peroxide production. *Adv. Mater.* **34**, 2107480 (2022).
16. Shiraishi, Y., Matsumoto, M., Ichikawa, S., Tanaka, S. & Hirai, T. Polythiophene-doped resorcinol–formaldehyde resin photocatalysts for solar-to-hydrogen peroxide energy conversion. *J. Am. Chem. Soc.* **143**, 12590-12599 (2021).
17. Zeng, X. *et al.* Simultaneously tuning charge separation and oxygen reduction pathway on graphitic carbon nitride by polyethylenimine for boosted photocatalytic hydrogen peroxide production. *ACS Catal.* **10**, 3697-3706 (2020).
18. Teng, Z. *et al.* Atomically dispersed antimony on carbon nitride for the artificial photosynthesis of hydrogen peroxide. *Nat. Catal.* **4**, 374-384 (2021).
19. Shiraishi, Y. *et al.* Resorcinol–formaldehyde resins as metal-free semiconductor photocatalysts for solar-to-hydrogen peroxide energy conversion. *Nat. Mater.* **18**, 985-993 (2019).
20. Chen, L. *et al.* Acetylene and diacetylene functionalized covalent triazine frameworks as metal-free photocatalysts for hydrogen peroxide production: a new two-electron water oxidation pathway. *Adv. Mater.* **32**, 1904433 (2020).

21. Kofuji, Y. *et al.* Hydrogen peroxide production on a carbon nitride–boron nitride-reduced graphene oxide hybrid photocatalyst under visible light. *ChemCatChem.* **10**, 2070-2077 (2018).
22. Kofuji, Y. *et al.* Carbon nitride–aromatic diimide–graphene nanohybrids: metal-free photocatalysts for solar-to-hydrogen peroxide energy conversion with 0.2% efficiency. *J. Am. Chem. Soc.* **138**, 10019-10025 (2016).
23. Kofuji, Y. *et al.* Graphitic carbon nitride doped with biphenyl diimide: efficient photocatalyst for hydrogen peroxide production from water and molecular oxygen by sunlight. *ACS Catal.* **6**, 7021-7029 (2016).
24. Du, J. *et al.* High-performance all-polymer solar cells: synthesis of polymer acceptor by a random ternary copolymerization strategy. *Angew. Chem. Int. Ed.* **59**, 15181-15185 (2020).
25. Lyu, W. *et al.* Fabrication of polyaniline/poly(vinyl alcohol)/montmorillonite hybrid aerogels toward efficient adsorption of organic dye pollutants. *J. Hazard. Mater.* **435**, 129004 (2022).
26. Zhang, Y. *et al.* Create a strong internal electric-field on PDI photocatalysts for boosting phenols degradation via preferentially exposing π-conjugated planes up to 100%. *Appl. Catal. B Environ.* **300**, 120762 (2022).
27. Han, W.-K. *et al.* Targeted construction of a three-dimensional metal covalent organic framework with spn topology for photocatalytic hydrogen peroxide production. *Chem. Eng. J.* **449**, 137802 (2022).
28. Zhang, F. *et al.* High-efficiency electrosynthesis of hydrogen peroxide from oxygen reduction enabled by a tungsten single atom catalyst with unique terdentate N_1O_2 coordination. *Adv. Funct. Mater.* **32**, 2110224 (2022).
29. Kresse, G. & Furthmüller, J. Efficient iterative schemes for ab initio total-energy calculations using a plane-wave basis set. *Phys. Rev. B.* **54**, 11169-11186 (1996).
30. Kresse, G. & Furthmüller, J. Efficiency of ab-initio total energy calculations for metals and semiconductors using a plane-wave basis set. *Comput. Mater. Sci.* **6**, 15-50 (1996).
31. Grimme, S., Antony, J., Ehrlich, S. & Krieg, H. A consistent and accurate ab initio parametrization of density functional dispersion correction (DFT-D) for the 94 elements H-Pu. *J. Chem. Phys.* **132**, 154104 (2010).
32. Blöchl, P. E. Projector augmented-wave method. *Phys. Rev. B.* **50**, 17953-17979 (1994).
33. Perdew, J. P., Burke, K. & Ernzerhof, M. Generalized gradient approximation made simple. *Phys. Rev. Lett.* **77**, 3865-3868 (1996).
34. Yanai, T., Tew, D. P. & Handy, N. C. A new hybrid exchange–correlation functional using the coulomb-attenuating method (CAM-B3LYP). *Chem. Phys. Lett.* **393**, 51-57 (2004).
35. Hariharan, P. C. & Pople, J. A. The influence of polarization functions on molecular orbital hydrogenation energies. *Theor. Chim. Acta.* **28**, 213-222 (1973).
36. Casida, M. E., Jamorski, C., Casida, K. C. & Salahub, D. R. Molecular excitation energies to high-lying bound states from time-dependent density-functional response theory: characterization and correction of the time-dependent local density approximation ionization threshold. *J. Chem. Phys.* **108**, 4439-4449 (1998).
37. Stratmann, R. E., Scuseria, G. E. & Frisch, M. J. An efficient implementation of time-dependent density-functional theory for the calculation of excitation energies of large molecules. *J. Chem. Phys.* **109**, 8218-8224 (1998).
38. Frisch, M. J. *et al.* Gaussian 16, Revision A.03 (Gaussian, Inc., Wallingford, CT, 2016).
39. Humphrey, W., Dalke, A. & Schulten, K. VMD: Visual molecular dynamics. *J. Mol. Graphics.* **14**, 33-38 (1996).

40. Lu, T. & Chen, F. Multiwfn: A multifunctional wavefunction analyzer. *J. Comput. Chem.* **33**, 580-592 (2012).
